# Characterization of an Atypical eIF4E Ortholog in *Leishmania*, LeishIF4E-6

**DOI:** 10.3390/ijms222312720

**Published:** 2021-11-24

**Authors:** Nitin Tupperwar, Rohit Shrivastava, Nofar Baron, Orli Korchev, Irit Dahan, Michal Shapira

**Affiliations:** 1 Department of Life Sciences, Ben-Gurion University of the Negev, Beer-Sheva 84105, Israel; nitinvet@gmail.com (N.T.); biorohit@gmail.com (R.S.); baronn@post.bgu.ac.il (N.B.); orlikor@post.bgu.ac.il (O.K.); orr@bgu.ac.il (I.D.); 2 CSIR-Centre for Cellular and Molecular Biology, Hyderabad 500007, India; 3 Centre de Recherche en Biologie Cellulaire de Montpellier, CNRS, 34090 Montpellier, France

**Keywords:** *Leishmania*, protein synthesis, translation regulation, LeishIF4E-6, LeishIF4G

## Abstract

*Leishmania* parasites are digenetic protists that shuffle between sand fly vectors and mammalian hosts, transforming from flagellated extracellular promastigotes that reside within the intestinal tract of female sand flies to the obligatory intracellular and non-motile amastigotes within mammalian macrophages. Stage differentiation is regulated mainly by post-transcriptional mechanisms, including translation regulation. *Leishmania* parasites encode six different cap-binding proteins, LeishIF4E1-6, that show poor conservation with their counterparts from higher eukaryotes and among themselves. In view of the changing host milieu encountered throughout their life cycle, we propose that each LeishIF4E has a unique role, although these functions may be difficult to determine. Here we characterize LeishIF4E-6, a unique eIF4E ortholog that does not readily associate with m^7^GTP cap in either of the tested life forms of the parasite. We discuss the potential effect of substituting two essential tryptophan residues in the cap-binding pocket, expected to be involved in the cap-binding activity, as judged from structural studies in the mammalian eIF4E. LeishIF4E-6 binds to LeishIF4G-5, one of the five eIF4G candidates in *Leishmania*. However, despite this binding, LeishIF4E-6 does not appear to function as a translation factor. Its episomal overexpression causes a general reduction in the global activity of protein synthesis, which was not observed in the hemizygous deletion mutant generated by CRISPR-Cas9. This genetic profile suggests that LeishIF4E-6 has a repressive role. The interactome of LeishIF4E-6 highlights proteins involved in RNA metabolism such as the P-body marker DHH1, PUF1 and an mRNA-decapping enzyme that is homologous to the TbALPH1.

## 1. Introduction

Trypanosomatid parasites are protozoans that alternate between insect vectors and mammalian hosts to complete their life cycle [1]. During their digenetic life cycle, *Leishmania* are found as extracellular promastigotes in the intestinal tract of female sand flies [2,3] and as the obligatory intracellular amastigotes within the macrophages of mammalian hosts [4]. This transition exposes the parasites to dramatic changes in their environmental conditions, which serve as signals to induce the developmental program of gene expression and stage differentiation. In higher eukaryotes, changes in environmental conditions induce a stress response that halts cap-dependent global protein synthesis while stress-response genes continue to be translated in a cap-independent manner [5,6]. However, exposure to the environmental switches described for *Leishmania* are an integral part of the parasite life cycle.

As with other Trypanosomatids, regulation of protein coding genes in *Leishmania* follows unique molecular features [7,8]. The protein-coding genes are arranged in large genomic clusters that are transcribed by RNA polymerase II to generate polycistronic mRNAs [9,10]. These mRNAs are further processed by trans-splicing and polyadenylation, to produce 5′capped and 3′poly (A) tailed mature mRNAs [11,12,13]. In the absence of conventional mechanisms for transcriptional activation of protein-coding genes, mRNA processing, stability and translation drive the stage-specific program of gene expression [8,9,14].

Translation initiation is a critical step in regulating protein synthesis in eukaryotes [15]. The canonical pathway includes the formation of a pre-initiation complex that contains a cap-binding complex, eIF4F, that consists of an mRNA cap-binding protein eIF4E; a large scaffold protein eIF4G and an RNA helicase, eIF4A. The scaffold eIF4G recruits the poly (A) binding protein (PABP) to the 3′ poly (A) chain of the mRNA, leading to mRNA circularization [16]. The association of the mRNA-bound eIF4F complex with other translation initiation factors together with the small ribosomal subunit complex scans the 5′ UTR until it reaches the first AUG, where translation initiates.

The *Leishmania* genome encodes six paralogs of the cap-binding protein eIF4E and five paralogs of eIF4G [17,18,19,20,21,22,23,24,25,26,27,28,29]. While the yeast genome encodes a single eIF4E that is essential for viability and two eIF4G homologs [30,31], mammalian cells encode for three eIF4Es and two eIF4Gs isoforms. The nematode *Caenorhabditis elegans* encodes five eIF4E isoforms which possess variable affinities towards the m^7^GTP and m^2,2,7^GTP cap analogs [32,33]. Of the multitude of eIF4E isoforms in *C. elegans*, not all play a role in canonical translation initiation and some are speculated to play a role as competitive inhibitors of eIF4G recruitment [34].

The six eIF4Es of *Leishmania* show poor sequence conservation with the eukaryotic eIF4Es or among themselves [18]. This possibly reflects duplication events that occurred during evolution, adapting each paralog to cover discrete functions during the life cycle of the parasite [35]. Analysis of the six *Leishmania* eIF4E paralogs showed that LeishIF4E-4 binds to the cap analogs only during the promastigote stage and forms a canonical cap-binding complex with LeishIF4G-3 and LeishIF4A-1 [18,20,21].

LeishIF4E-3 is a weak cap-binding protein but interacts with LeishIF4G-4 under normal conditions. However, during nutritional stress, also experienced during metacyclogenesis, the two proteins dissociate and LeishIF4E-3 enters into starvation-induced RNA granules [36]. LeishIF4E-3 is essential in trypanosomatids and deletion of a single allele affected global translation and parasite infectivity, suggesting that LeishIF4E-3 has an active role in global protein synthesis under normal conditions [25,37].

LeishIF4E-2 is one of the least studied 4E paralogs in *Leishmania*. It does not interact with any of the LeishIF4Gs, but migrates with the heavy polysome complexes in sucrose gradients, possibly stabilizing polysome complexes [18]. Hemizygous deletion of LeishIF4E-2 does not affect global translation and growth rate but affects the morphology of promastigotes, possibly by changes in specific cytoskeleton proteins [23]. Thus, LeishIF4E-2 does not appear to function as a general translation initiation factor and hence its function remains obscure.

The only LeishIF4E paralog that retains its cap-binding activity in axenic amastigotes is LeishIF4E-1. LeishIF4E-1 expression increases during metacyclogenesis and in amastigotes and maintains its cap-binding activity throughout these life stages. Although it does not interact with any of the LeishIF4G candidates in *Leishmania*, it was shown to be involved in active translation [38]. This was established by CRISPR-Cas9 mediated deletion that was marked by a reduction in global translation and changes in the promastigote specific proteome [38]. The LeishIF4E-1 function is regulated by two LeishIF4E-1 interacting proteins, 4E-IP1 and 4E-IP2 [21,39]. The tight interaction of LeishIF4E-1 with its regulatory protein 4E-IP1 was verified by structural studies as well [40].

Two additional isoforms of eIF4E have been identified in *Trypanosoma brucei*, TbIF4E-5 and TbIF4E-6 [41,42]. Recently, we investigated the *Leishmania* ortholog of TbIF4E-5. LeishIF4E-5 binds weakly to the m^7^GTP cap analog, but only during the promastigote stage. Mass spectrometry analysis of proteins that co-purify with LeishIF4E-5 highlighted proteins involved in RNA metabolism, along with two LeishIF4G paralogs, LeishIF4G-1 and LeishIF4G-2. Overexpression of LeishIF4E-5 showed a decline in cell proliferation and an overall reduction in global translation. Thus, LeishIF4E-5 could be a putative translational repressor [24].

Here, we investigate LeishIF4E-6, the *Leishmania* ortholog of TbIF4E-6. We show that it has no affinity to the m^7^GTP cap analog, and in silico analysis provides a structural explanation to this observation. Overexpression of LeishIF4E-6 in transgenic parasites reduces global translation, excluding the possibility that LeishIF4E-6 is a canonical translation factor and possibly suggesting that it has a repressive role. The hemizygous deletion of LeishIF4E-6 affected the morphology of promastigotes, in a manner not yet understood.

## 2. Results

### 2.1. Sequence Analysis of LeishIF4E-6 and Secondary Structure Prediction

**Figure 1:**
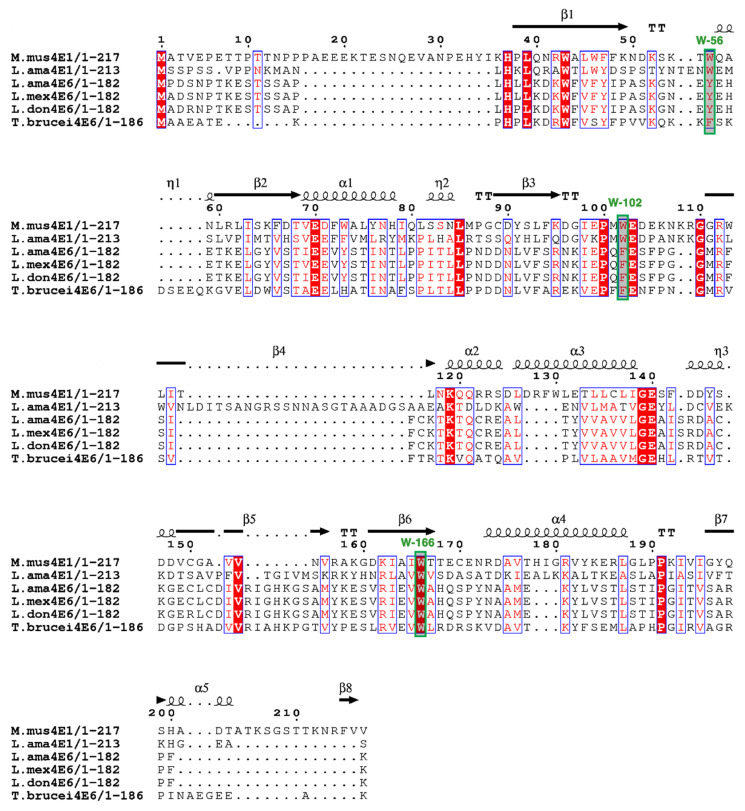
Multiple sequence alignment of the LeishIF4E-6. The open reading frames of eIF4E-6 from different *Leishmania* species and *T. brucei* along with mammalian and *L. amazonensis* LeishIF4E-1 were aligned using Jalview (2.10.5). The sequences were retrieved from *Mus musculus* eIF4E-1 (ENSMUSG00000028156), *L. amazonensis* LeishIF4E-1 (LAMA_000544700), *L. amazonensis* LeishIF4E-6 (LAMA_000504800); *L. mexicana* LeishIF4E-6 (LmxM.26.0240) *L. donovani* LeishIF4E-6 (LdBPK.26.2.000230) and *T. brucei* TbIF4E-6 (Tb927.7.1670). The alignment file was saved in FASTA format. The final alignment showing the predicted secondary structure was developed using the downloaded PDB file (6YLT, doi:10.2210/pdb6YLT/pdb) of *Mus musculus* eIF4E-1 (https://www.rcsb.org/structure/6YLT, accessed on 5 July 2021) along with the FASTA alignment file, using the online ESPript 3 tool. Symbols for the secondary structure elements were (α: alpha helices, ƞ: 3_10_-helix, β: beta-strands, TT: strict β-turns). White letters over a red background correspond to identical residues while red letters over a white background correspond to similar residues.

Protein sequences of eIF4E-6 from various *Leishmania* and *Trypanosoma* species were aligned by Jalview (2.10.5) and further subjected to a secondary structure alignment by using the 3D structure of *Mus musculus* eIF4E1 from PDB (https://www.rcsb.org/) (Figure 1). LeishIF4E-6 shows 31.4% similarity with the mouse eIF4E. The LeishIF4E-6 amino acid sequence differs slightly from that of the *T. brucei* ortholog (Figure S1A,B). Among the various LeishIF4Es, the amino acid sequence of LeishIF4E-6 shows the highest similarity with LeishIF4E-5 (43.2%) and a lower similarity with other LeishIF4Es, ranging between 18.7–27.3% (Figure S1B). We used the online ESPript 3 tool along with the already generated FASTA alignment file to make the final secondary structure prediction. The predicted structure of LeishIF4E-6 is mostly enriched with alpha-helices, beta-strands and beta-turns; however, the overall structure is conserved among different eIF4E-6 sequences from *Leishmania* and *Trypanosoma* species (Figure 1). The typical cap-binding pocket of mammalian eIF4E contains the three conserved Tryptophans at positions 56, 102, and 166 [43]. The TbIF4E-6 and *L. amazonensis* LeishIF4E-6 proteins have a Trp residue at the position that is analogous to the mouse Trp 166, while both have a Phe at the position that corresponds to Trp 102 of the mammalian eIF4E. The corresponding position of Trp 56 in the mammalian eIF4E is occupied by Tyr and Phe residues in LeishIF4E-6 and TbIF4E-6, respectively (Figure 1 and Figure S1A).

### 2.2. LeishIF4E-6 Is Localized in the Cytoplasm

**Figure 2:**
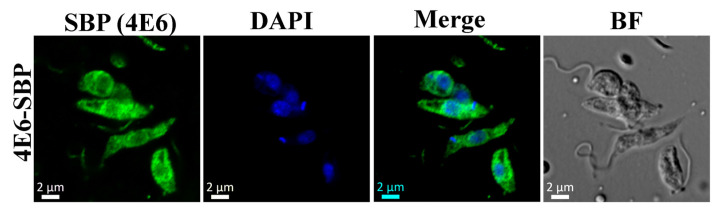
SBP tagged LeishIF4E-6 localizes in the cytoplasm. Transgenic *L. amazonensis* promastigotes overexpressing SBP-tagged LeishIF4E-6 were fixed and processed for confocal microscopy. LeishIF4E-6-SBP was detected using a monoclonal antibody against the SBP tag and secondary DyLight-labeled antibodies (488 nm; green, SBP). Nuclear and kinetoplast DNA was stained using DAPI (blue). A bright field (BF) picture of the cells is shown on the right. The confocal analysis was repeated three times.

The subcellular localization of a protein can serve as a preliminary lead on its potential function. Transgenic *Leishmania* cells overexpressing the C-terminal SBP-tagged LeishIF4E-6 were therefore used to examine the intracellular localization of LeishIF4E-6 by confocal microscopy using antibodies against the SBP tag. Figure 2 shows that LeishIF4E-6-SBP was non-uniformly distributed in the cytoplasm with the protein stained in patches of strong cytoplasmic puncta. We also compared this to the localization of TbIF4E-6 in the TrypTag database. The TrypTag is a database that is aimed at curating the localization of every protein in *Trypanosoma brucei* using mNeonGreen as a fluorescent tag. In that case too, we observed that TbIF4E-6 shows a patchy cytoplasmic localization as well, with 25% of the protein localized in the nucleolus [44].

### 2.3. Over-Expression of LeishIF4E-6 Reduced Global Translation

**Figure 3:**
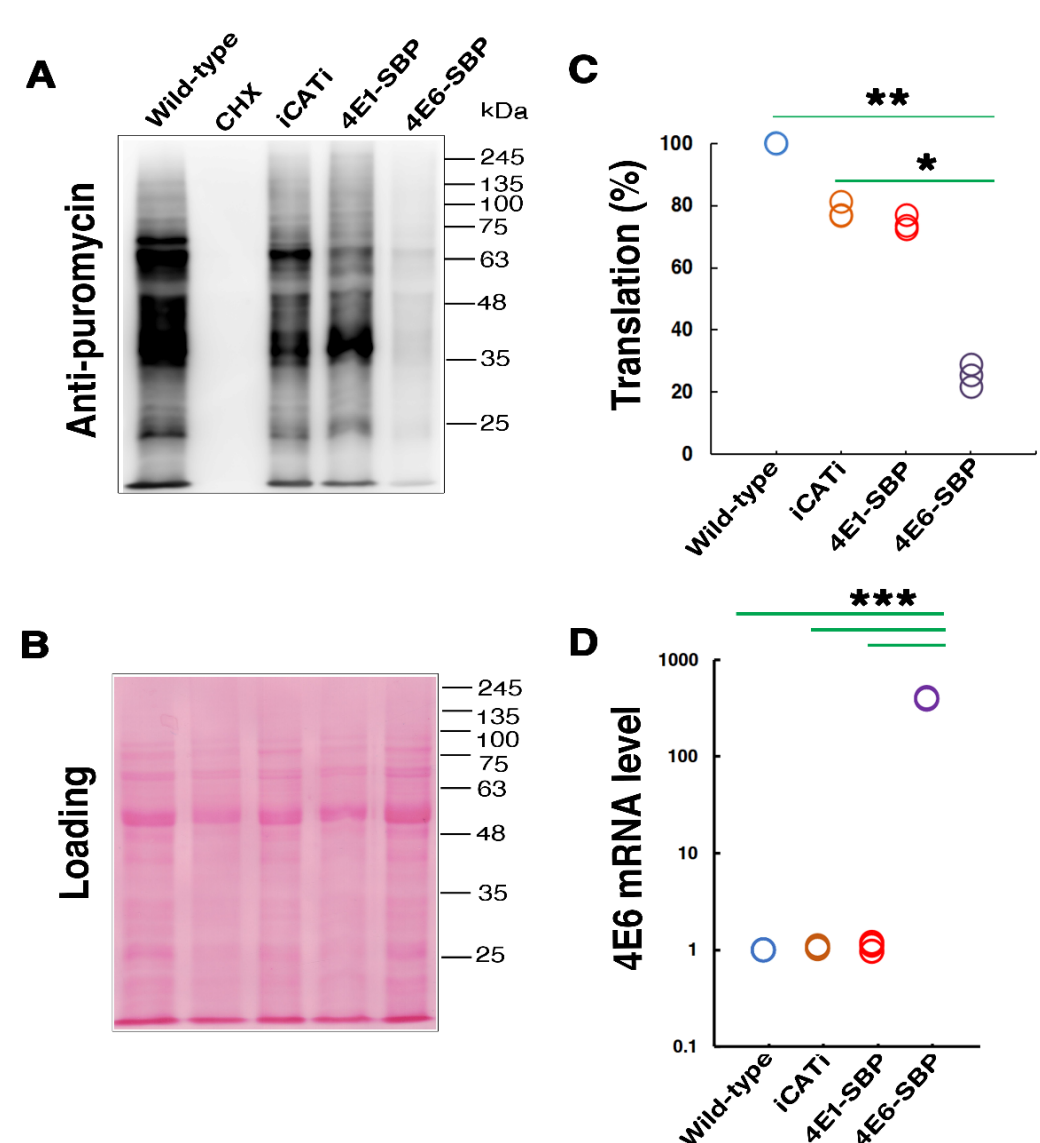
Overexpression of LeishIF4E-6 reduces global translation. Transgenic *L. amazonensis* promastigotes overexpressing the SBP-tagged LeishIF4E-6 and LeishIF4E-1, the Chloramphenicol acetyltransferase (CAT) reporter, along with wild type *L. amazonensis*, were examined in the SuNSET assay. (**A**) Cells were incubated with 1 µg/mL puromycin for 20 min, extracted, separated over 12% SDS-PAGE and subjected to western analysis using specific antibodies against puromycin. A cycloheximide (CHX) control for complete translation inhibition is also shown. Whole cell extracts were separated by 12% SDS-PAGE and subjected to western analysis using antibodies against puromycin. (**B**) Ponceau staining of the blot shows comparable protein loads; (**C**) densitometry data (taken with Multi-Gauge, version 2.0) quantify the puromycin incorporation in the different cell lines, as compared to puromycin incorporation in wild type cells (100%). GraphPad Prism was used for statistical analysis. Three independent experiments were performed and the individual values are presented as dots. Statistical significance was determined using a Kruskal–Wallis test with Dunn’s multiple-comparison test for comparing three or more groups. Significant *p*-values were marked as follows: *p*-value < 0.05 (*), *p*-value < 0.01 (**). (**D**) LeishIF4E-6 transcript levels in the different cell lines were determined by quantitative real-time PCR. The relative expression of the LeishIF4E-6 transcripts was calculated using the 2^−ΔΔC^_T_ formula where the endogenous control GAPDH was used as the normalizer. The values of three replicates were represented as a dot plot graph. The statistical analysis was carried out using GraphPad Prism 5 and significant differences were evaluated by the non-parametric T-test using the paired *t*-test. Significant *p*-values were marked as follows: *p*-value < 0.001 (***).

Cap-binding proteins can function as activators or repressors of translation. To examine the potential role of LeishIF4E-6, we tested how its overexpression affected global translation. The global translation was evaluated by the SUnSET assay in transgenic *L. amazonensis* cells overexpressing the SBP-tagged LeishIF4E-6. Control lines expressed the SBP-tagged LeishIF4E-1, the Chloramphenicol acetyltransferase (CAT) reporter protein flanked by HSP83 intergenic regions (i), and wild-type (WT) parasites. The SUnSET assay is based on the incorporation of puromycin into the A site of the ribosome, as puromycin is a structural analog of aminoacyl-tRNA. The incorporated puromycin results in the termination of polypeptide synthesis. Total extracts of the treated cells are blotted onto nitrocellulose membranes that are further incubated with antibodies against puromycin. The amount of puromycin incorporated represents the total translation events at a given time point. Figure 3A shows that LeishIF4E-6 overexpressing cells show a reduction in their global translation as compared to the control cell lines that overexpress LeishIF4E-1 and iCATi as well as wild-type cells. Figure 3B shows the Ponceau staining representing comparable protein loads which is important for quantification of translation by densitometry analysis. The global translation in LeishIF4E-6 overexpressing cells was significantly reduced to ~25% of the translation level measured in the wild-type cells that were set as 100%. Total translation in the cell lines overexpressing LeishIF4E-1 and iCATi was ~74% and ~78% of the wild-type control, respectively (Figure 3C). We have earlier reported that cell lines overexpressing transfected exogenous genes show a reduction in their total translation as compared to wild-type cells [23,38]. This reduction could be due to the competition for components of the translation machinery for the synthesis of overexpressed proteins. However, the total translation in LeishIF4E-6 overexpressing cells was reduced to a much lower level as compared to control cells, suggesting that overexpression of LeishIF4E-6 could have a repressive effect on translation.

To monitor overexpression of LeishIF4E-6 in the transgenic cell lines, the LeishIF4E-6 transcript levels in different cell lines were examined by quantitative real-time PCR assay. The relative expressions of the LeishIF4E-6 transcripts in different cell lines were calculated using the 2^−ΔΔCT^ formula in which the glyceraldehyde 3-phosphate dehydrogenase gene (GAPDH) served as the endogenous control. The LeishIF4E-6 transcript levels in cells transfected with an episome carrying the LeishIF4E-6-SBP gene increased by 400 fold as compared to the LeishIF4E-6 level in wild type (WT) cells and in the other control cell lines (Figure 3D and Table S4).

### 2.4. LeishIF4E-6 Does Not Associate with the m^7^GTP Cap Analog in Promastigotes and Axenic Amastigotes

**Figure 4:**
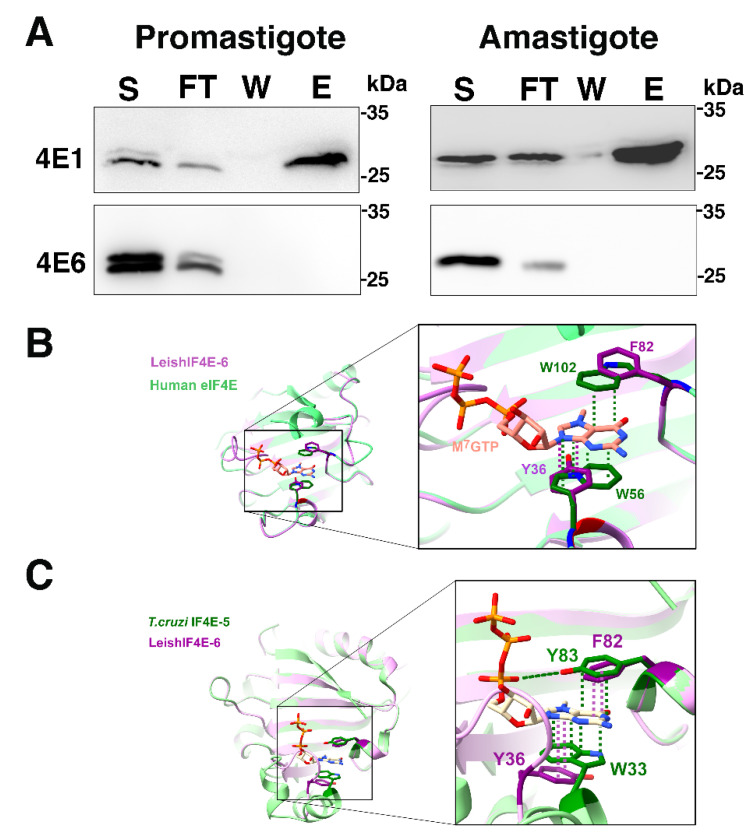
(**A**) LeishIF4E-6 does not bind the cap analog m^7^GTP in promastigotes and axenic amastigotes. Lysates of SBP-tagged LeishIF4E-6 overexpressing cells were obtained from promastigotes and axenic amastigotes. The lysates were incubated with m^7^GTP-agarose beads that were washed and further eluted with free m^7^GTP (200 μM). The eluted proteins and wash fractions were precipitated with 10% TCA for further analysis over SDS-PAGE (12%). The gels were loaded with samples from the total supernatants (S, 2%), flow-through fractions (FT, 2%), the final wash fractions (W, 50%) and the eluted fractions (E, 50%). The blots were analyzed using monoclonal antibodies against the SBP-tag (fused to LeishIF4E-6) and against LeishIF4E-1 that served as a positive control for m^7^GTP binding in the same extracts. Similar results were obtained from three independent experiments; (**B**) structural differences in the predicted m^7^GTP binding pocket between the human eIF4E and LeishIF4E-6. The LeishIF4E-6 homology model (purple) was superposed on the human eIF4E (PDB: 5T46, green). The different amino acids in the cap-binding pocket are represented as sticks, with oxygen atoms in red, nitrogen in blue and phosphates in orange. The π stacking interactions between m^7^GTP and the aromatic residues are shown as dashed lines in colors that correspond to the aromatic residues from the different organisms (human in green and *Leishmania* in purple); (**C**) structural differences in the predicted m^7^GTP binding pocket between the LeishIF4E-6 and *T. cruzi* IF4E-5. The LeishIF4E-6 homology model (purple) was superposed on the *T. cruzi* IF4E-5 (PDB: 6O80, green). The different amino acids in the cap- binding pocket are represented as sticks, oxygen in red, nitrogen in blue and phosphate in orange. The π stacking interactions between m^7^GTP to the aromatic residues are shown as square dotted lines in suitable colors. The hydrogen bond between Y83 (green) and the m^7^GTP phosphate oxygen is represented by elongated dotted lines in the suitable color.

LeishIF4E-6-SBP overexpressing cells showed a reduction in their global translation (Figure 3), indicating that overexpression of LeishIF4E-6 could have a potential repressive effect on translation. To further examine its function, we monitored the cap-binding activity of LeishIF4E-6 in promastigotes and axenic amastigotes. Functional m^7^GTP pull-down assay was carried out with extracts of mid-log phase cells overexpressing LeishIF4E-6-SBP. Cells that expressed LeishIF4E-1-SBP were used as a positive control for binding to m^7^GTP in both life stages of the parasite [21] showing that the cells were functional, also supporting that the C-terminal tag did not prevent m^7^GTP binding. The different cell extracts were loaded onto m^7^GTP -agarose beads that were washed and eluted with free m^7^GTP. Aliquots from the supernatant, flow-through, wash, and the elution fractions were resolved over SDS-PAGE gel and further subjected to western analysis with antibodies against the SBP-tag and LeishIF4E-1. Figure 4A shows that LeishIF4E-6 failed to bind to the m^7^GTP cap in both the stages of the parasite, while the binding of LeishIF4E-1 to m^7^GTP that served as the positive control was very efficient.

The cap-binding pocket in the human eIF4E contains three conserved Trp residues at positions 56, 102 and 166 [43]. Trp56 and Trp102 create a π sandwich interaction with the guanine ring that stabilizes its binding. However, in LeishIF4E-6, these residues are replaced by Tyr36 and Phe82, respectively (Figure 4B). Although the substituted residues are aromatic, these changes could weaken the π interaction between Phe82 and Tyr36 with the methylated guanine of m^7^GTP, thus leading to a decreased affinity between the LeishIF4E-6 and the m^7^GTP. Structural comparison of the cap-binding pocket of LeishIF4E-6 to that of *T. cruzi* TcIF4E-5 [45] also indicates that the aromatic residues are substituted in a manner that could weaken the π interactions with m^7^GTP, with Tyr83 and Trp33 in TcIF4E-5 exchanged with Phe82 and Tyr36, respectively, in LeishIF4E-6 (Figure 4C). The inability of LeishIF4E-6 to bind m^7^GTP remains an intriguing observation that is partly unexplained. The replacement of the conserved Trp residues in the cap-binding pocket could impair such binding, but we expect that other structural features not yet identified could have contributed to this inability to bind the cap structure.

### 2.5. The LeishIF4E-6 Interactome

The presence of multiple eIF4E paralogs that form unique cap-binding complexes during the life cycle of *Leishmania* could indicate that they are responsible for unique functions. The interacting proteins that are part of the LeishIF4E-6 complex were identified in cells that express SBP-tagged LeishIF4E-6. Cell extracts were affinity-purified over streptavidin-Sepharose beads, washed and eluted with biotin. The eluted fractions were first examined by western analysis to verify that the bait protein was eluted, and then analyzed by LC-MS/MS. Cells overexpressing SBP tagged Luciferase were used as a negative control.

**Figure 5:**
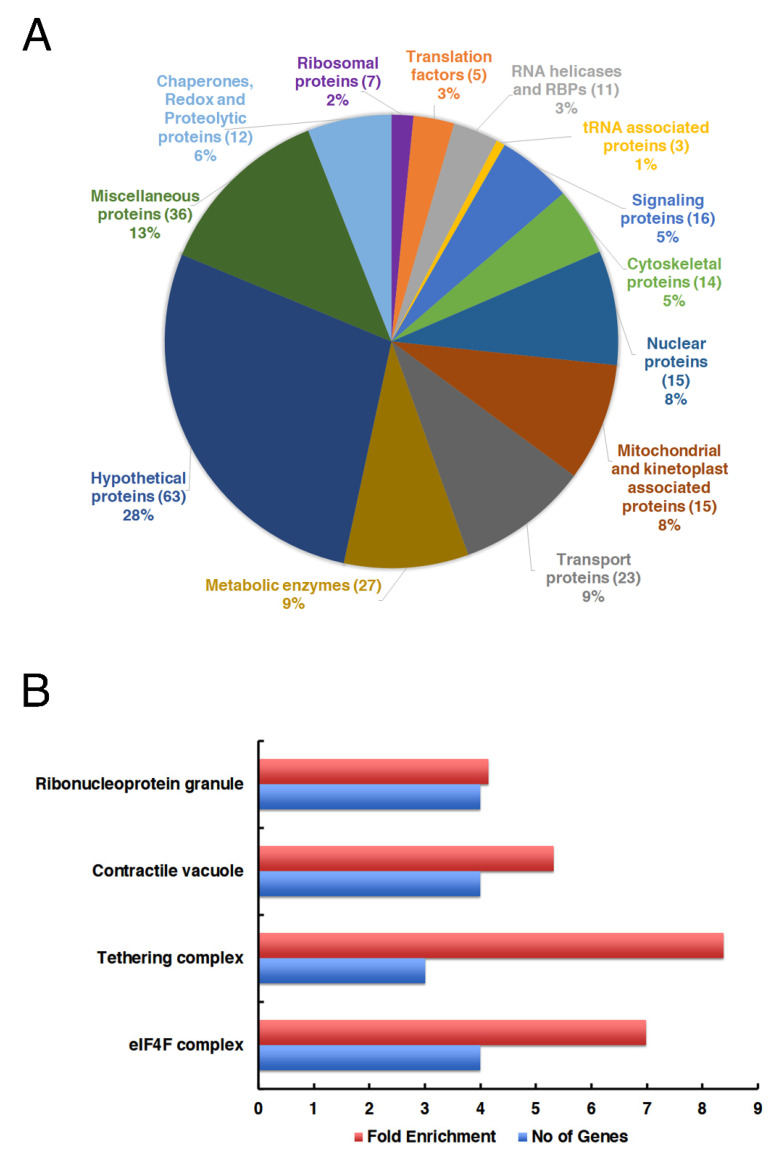
The proteomic content of LeishIF4E-6 associated proteins. Extracts of the cells overexpressing SBP-tagged LeishIF4E-6 were purified over streptavidin beads, washed and eluted with biotin. The eluted proteins were subjected to LC-MS/MS analysis. MaxQuant software was used to identify the detected proteins. Luciferase overexpressing cells were used as a negative control to subtract the background proteins. Perseus statistical tool was used to set a cut-off value of 1.6 Log_2_ fold (three-fold change) and a *p*-value < 0.05. (**A**) The pie chart represents the manually categorized clusters of proteins that were enriched in the LeishIF4E-6 associated fraction, as compared to the luciferase control. (**B**) Proteins that were enriched in the LeishIF4E-6 associated fraction were subjected to the Gene Ontology enrichment tool in TryTripDB based on cellular components. The threshold value for GO term was set to 4-fold with *p*-value < 0.01.

Proteins that were pulled down from the SBP-tagged LeishIF4E-6 and control luciferase were identified by LC-MS-MS, and the peptides were compared to the *L. major* protein database in TriTrypDB [46] using the MaxQuant software. Statistical analysis was performed using the Perseus software to identify proteins that were enriched in the LeishIF4E-6 pull-down fraction, as compared to the luciferase control. We set a cut-off value at Log_2_ fold of 1.6 (three-fold change) and a *p*-value < 0.05. Figure 5A and Table S1 show the enriched proteins in the LeishIF4E-6-SBP interactome, following their manual clustering into different functional categories.

The manual categorization of the LeishIF4E-6-SBP pull-down analysis showed significant enrichment of 238 proteins that were related to different cellular pathways, including translation, metabolism, transport and signaling, as well as proteins defined as nuclear or mitochondrial. The LeishIF4E-6 interactome also contained ribosomal and several RNA-binding proteins. It was previously discussed that metabolic enzymes can bind to RNA even if they do not contain the canonical RNA binding domains, and these could therefore be purified along with the LeishIF4E-6 interactome [47]. We also assume that metabolic enzymes could serve as sensors for the metabolic status of the cells, thus affecting translation in a manner not yet resolved. Among the translation factors, we noticed a strong association of LeishIF4E-6 with the LeishIF4G-5 scaffold protein (Figure 5A).

The manual categorization of the LeishIF4E-6 interactome by LC-MS-MS analysis showed that LeishIF4E-6 co-purified with one of the five LeishIF4G paralogs, LeishIF4G-5. The co-purification of the TbIF4G-5 scaffold protein with TbIF4E-6 was also observed in *T. brucei*, showing that these two proteins form a specific complex. Sequence analysis of LeishIF4G-5 highlights two Y(X)_4_LΦ motifs, which are common in most of the eIF4E-binding proteins (Figure S2A). InterPro domain analysis [InterPro(ebi.ac.uk)] of LeishIF4G-5 sequence detected the presence of a MIF4G domain that is present in most of the LeishIF4Gs (Figure S2B). LeishIF4E-6 also co-purified with a homolog of TbG5-IP (LmjF.32.1370), which was shown to be associated with TbIF4G-5.

The proteins that were associated with LeishIF4E-6 were further subjected to the Gene Ontology (GO) enrichment analysis, based on the cellular components (Figure 5B and Table S2). The threshold value for the GO term was set to 4-fold with a *p*-value < 0.01. The GO enrichment analysis highlighted proteins associated with ribonucleoprotein granules including DHH1, a *Leishmania* ortholog of the decapping enzyme TbALPH1 [48], LmjF.22.1600, and translation factors that constitute the eIF4F complex. Other groups that were highlighted by the GO analysis included proteins that belong to the tethering complex and contracting vacuoles (Figure 5B and Table S2), suggesting that LeishIF4E-6 could interact with cytosolic granules and vacuoles. The association of LeishIF4E-6 with ribonucleoprotein proteins, the granule-specific ATP-dependent RNA helicases (DHH1) and RNA binding proteins such as (PUF-1 and the Alba-domain protein 1) indicate their roles in the RNA metabolism (Table S1). In addition, GO term analysis highlighted the presence of tethering complex proteins involved in vesicular transport (COG6, Exo99, TRAPPC) and contractile vacuole complex proteins (Calmodulin).

Sequence conservation among the different LeishIF4G candidates in *Leishmania* is low, and LeishIF4G-5 shows 20–30% similarity to other LeishIF4Gs in amino acid sequences and 19% similarity with *Mus musculus* eIF4G (Figure S2C).

### 2.6. LeishIF4E-6 Interacts Directly with LeishIF4G-5

**Figure 6:**
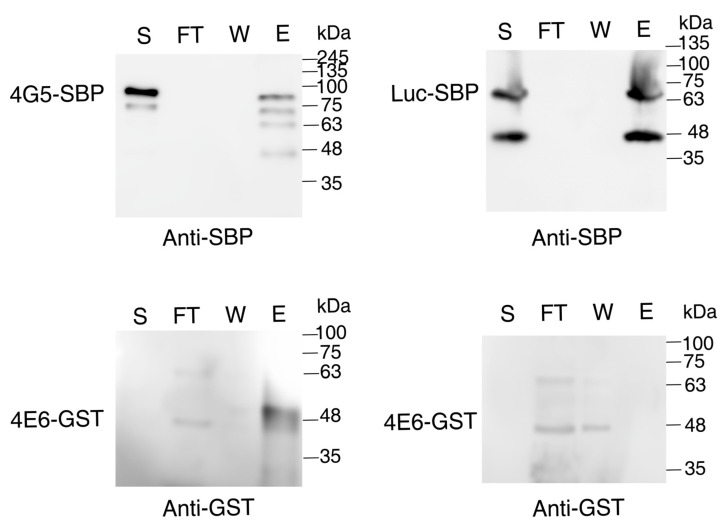
Recombinant GST-LeishIF4E-6 interacts directly with LeishIF4G-5. Lysates of transgenic *Leishmania* cell lines expressing the SBP tagged LeishIF4G-5 were bound to Streptavidin beads, washed and further incubated with the *E. coli* cell lysates expressing the recombinant GST tagged LeishIF4E-6 (left panels, top and bottom). After several washes, the beads were eluted with 5 mM Biotin. A *Leishmania* cell line expressing the transgenic SBP-tagged Luciferase was used as a negative control (right panels, top and bottom). All gels were loaded with aliquots obtained from supernatants of the cell extracts (S, 2%), the flow through (FT, 2%), the wash (W, 25%) and the eluted fractions (E, 25%) were resolved over 12% SDS-PAGE, which were subjected to Western Blot analysis using the specific monoclonal antibodies against the SBP and GST tags. The top panels represent the blot developed with anti-SBP antibodies while the bottom panels represent the blot developed with anti-GST antibodies.

We further demonstrated the direct interaction between LeishIF4G-5 and LeishIE-6 using recombinant GST-LeishIF4E-6 expressed in bacteria (Figure S3) and SBP-tagged LeishIF4G-5 expressed in *Leishmania*. SBP-tagged Luciferase was used as a negative control. Lysates of cells overexpressing LeishIF4G-5-SBP or Luciferase-SBP were bound to streptavidin-Sepharose beads, washed to remove unbound material, and further incubated with bacterial lysates containing recombinant GST-tagged LeishIF4E-6. Following several washes, the bound proteins were eluted by 5 mM Biotin. Aliquots from the different fractions including the supernatant, flow-through, wash, and eluted proteins were resolved on SDS-PAGE gels and subjected to western analysis using specific antibodies against the SBP and GST. LeishIF4G-5-SBP co-eluted with LeishIF4E-6-GST, whereas the negative control of Luciferase-SBP failed to co-elute LeishIF4E-6-GST, indicating that LeishIF4E-6 interacted directly with LeishIF4G-5 (Figure 6). We noticed several bands of LeishIF4G-5 in the western analysis that could originate from protein breakdown. Further confirmation that these multiple bands were derived from LeishIF4G-5 were obtained from mass spectrometry analysis performed on each of the cleavage products (Figure S4A,B and Table S3). Cleavage of proteins that are part of the translation apparatus is common in *Leishmania*, as previously discussed [24,39].

### 2.7. Hemizygous Deletion of LeishIF4E-6 by CRISPR-Cas9 Leads to a Defective Morphology of Promastigotes

**Figure 7:**
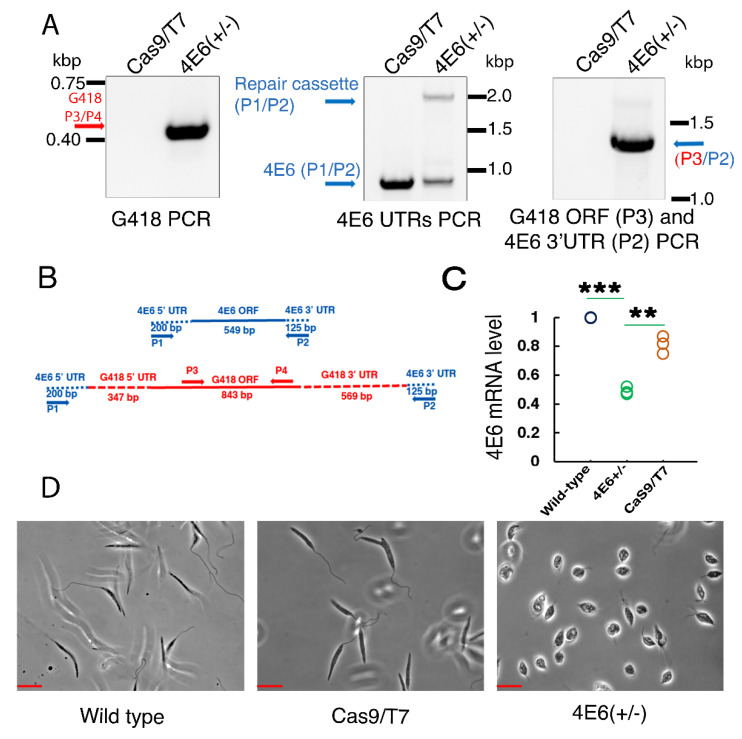
CRISPR-Cas9 mediated hemizygous deletion of LeishIF4E-6 affects cell morphology (**A**) Diagnostic PCR was performed to confirm the deletion of a single allele of LeishIF4E-6. Genomic DNA was extracted from *L*. *mexicana* Cas9/T7 cells and the LeishIF4E-6(+/−) mutant. PCR was performed using different combinations of primers derived from the G418 ORF (Forward) and Reverse (P3/P4, middle panel), LeishIF4E-6 5′ UTR (Forward) and 3′ UTR (Reverse, P1/P2 –left panel); and a primer set derived from the G418 resistance gene (forward) and the 3′ UTR (Reverse, P3/P2); (**B**) a schematic representation of the LeishIF4E-6 locus and the primers used (represented by arrows). The PCR was applied to test the presence or absence of the LeishIF4E-6 gene and the G418 resistance marker. Primers derived from the LeishIF4E-6 UTRs are shown in blue and primers derived from the ORF of G418 are shown in red. (**C**) The relative decrease in the LeishIF4E-6 transcript levels in hemizygous LeishIF4E-6(+/−) mutant was determined by quantitative real-time PCR assay. The relative expression of the LeishIF4E-6 transcripts was calculated using a 2^−ΔΔC^_T_ formula where endogenous control GAPDH was used as the normalizer. The values of three replicates were represented as a dot plot graph. The statistical analysis was carried out using GraphPad Prism 5 and significant differences were estimated by a non-parametric T-test using the paired *t*-test. Significant *p*-values were marked as follows: *p*-value < 0.01 (**) and *p*-value < 0.001 (***). (**D**) Mid-log phase (Day 2) promastigotes of WT, Cas9/T7 expressers, and LeishIF4E-6(+/−) cells were fixed with 2% paraformaldehyde and visualized by phase-contrast microscopy at 100x magnification. Wild-type, Cas9/T7 overexpressing cells showed normal promastigote morphology while LeishIF4E-6(+/−) were round swollen with reduced flagellar length. The red scale bar represents 10 µm.

To study the function of LeishIF4E-6, we attempted to delete it by CRISPR-Cas9. We used specific sgRNAs that targeted the 5’ and 3’ UTRs of LeishIF4E-6, resulting in the replacement of LeishIF4E-6 by the G418 or Blasticidin antibiotic repair fragments. Both sgRNAs, as well as G418 and Blasticidin repair fragments, were electroporated into active early mid-log cells. Cell lines in which the target gene was deleted were individually selected by their resistance to either G418 (200 µg/mL) or Blasticidin (20 µg/mL). Our attempt to screen for cells that were resistant to both drugs applied simultaneously did not generate viable cells. We obtained viable cells only in the presence of a single antibiotic, G418. The diagnostic PCR was set with genomic DNA extracted from the G418 resistant LeishIF4E-6 mutant and Cas9/T7 control cells, using different primer combinations. To verify the presence of the G418 selection marker in the genomic DNA of the mutant, we performed the PCR with the primers P3/P4 derived from the G418 resistance gene. The reaction yielded a 450 bp product exclusively with the gDNA from the deletion mutant LeishIF4E-6 but not with the Cas9/T7 control DNA (Figure 7A left panel). Primers derived from the 5′ and 3′ UTRs of the LeishIF4E-6 generated a PCR product of 874 bp with the Cas9/T7 control, and two products of 874 bp and 1991 bp with the mutant DNA, indicating a proper replacement of the target LeishIF4E-6 gene with the repair cassette (primers P1/P2, Figure 7A, middle panel), but one of the two endogenous alleles of LeishIF4E-6 gene was not eliminated. This indicated that we were able to delete only a single copy of the LeishIF4E-6. To further validate that the G418 repair fragment was incorporated at the proper target position that replaced the LeishIF4E-6 gene, the integration site was examined by a PCR using another primer set, P2/P3. P3 was derived from the G418 ORF, whereas P2 was derived from the 3′ UTR of LeishIF4E-6. A PCR product of 1353 bp was observed only in the deletion mutant of LeishIF4E-6 but not in the Cas9/T7 control (Figure 7A right panel and 7B). The results indicated the proper insertion of the G418 repair cassette at the target position and the removal of a single LeishIF4E-6 gene, generating a hemizygous mutant of LeishIF4E-6. Figure 7B shows the schematic representation of different primer positions. To monitor the reduction in LeishIF4E-6 expression in the hemizygous LeishIF4E-6(+/−) mutant, we performed a quantitative real-time PCR analysis. The relative expression of the LeishIF4E-6 transcripts was calculated using the 2^−ΔΔC^_T_ formula, where the endogenous control GAPDH transcript was used as the normalizer. This analysis revealed a ~50% reduction of the LeishIF4E-6 transcript levels in the hemizygous LeishIF4E-6(+/−) mutant (Figure 7C and Table S4).

We examined the promastigote morphology of the LeishIF4E-6(+/−) along with the control cell lines. We noticed a strong phenotypic change in the mutant. Control cells were typically elongated with large protruding flagella while the LeishIF4E-6(+/−) cells lost their typical promastigote morphology and became small, rounded up and swollen and, in addition, their flagellar length was reduced (Figure 7D).

### 2.8. Global Translation in Hemizygous Mutant LeishIF4E-6(+/−) Is Unaffected

**Figure 8:**
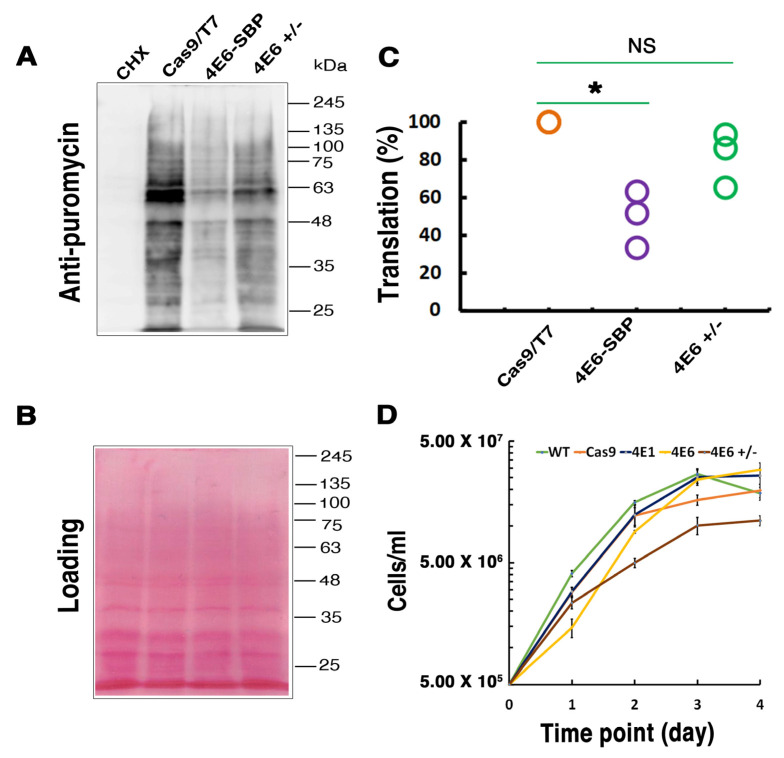
Global translation in the hemizygous mutant LeishIF4E-6(+/−). The SBP-tagged LeishIF4E-6 and the LeishIF4E-6(+/−) mutants along with Cas9/T7 overexpressing cells were used for the SUnSET assay. Cycloheximide treated wild type cells served as a negative control for translation. (**A**) All cells were then incubated with 1 µg/mL puromycin for 20 min, extracted, separated over 12% SDS-PAGE and subjected to western analysis using specific antibodies against puromycin. A cycloheximide (CHX) control for complete translation inhibition is also shown. (**B**) Similar protein loads were validated by Ponceau staining of the blots; (**C**) densitometry data to quantify the puromycin incorporation in the different cell lines, as compared to puromycin incorporation in Cas9/T7 overexpressing control cells (100%). GraphPad Prism was used for statistical analysis. Three independent experiments were performed, and the individual values are presented as dots. Statistical significance was determined using the Kruskal–Wallis test with Dunn’s multiple-comparison test for comparing three or more groups. Significant *p*-values were marked as follows: *p*-value < 0.05 (*). NS is non-significant. (**D**) Growth curves of the different cell lines were generated by monitoring the cell counts during five consecutive days. Wild-type cells are shown in green, cells overexpressing the Cas9/T7 are in orange, cells overexpressing LeishIF4E-1 are in blue, cells overexpressing LeishIF4E-6 are shown in yellow, and the hemizygous mutant LeishIF4E-6(+/−) cells are shown in brown.

To further validate the repressive role of LeishIF4E-6 in translation, we carried out the SUnSET assay using the hemizygous mutant of LeishIF4E-6. The assay was done on different *Leishmania* cell lines that consisted of the hemizygous LeishIF4E-6(+/−) mutant along with cells that overexpressed the LeishIF4E-6-SBP from an episomal vector, and Cas9/T7 overexpressing control cells. The LeishIF4E-6 overexpressing cell line showed a reduction in global translation; however, translation in the hemizygous mutant LeishIF4E-6(+/−) was stronger as compared to the LeishIF4E-6 overexpressing line (Figure 8A). Figure 8B shows the Ponceau staining of the blot, validating comparable protein loads. A decline of 18% in the global translation activity of the LeishIF4E-6(+/−) cells was observed when compared with Cas9/T7 control cells. However, the decline in the global translation of the LeishIF4E-6 overexpressing cells was higher (~50%, Figure 8C). We also monitored the cell growth of different transgenic cell lines overexpressing LeishIF4E-6-SBP, LeishIF4E-1-SBP, Cas9/T7, along with the LeishIF4E-6(+/−) hemizygous mutant and wild-type cells. We observed a growth delay in the LeishIF4E-6-SBP cells; however, their growth recovered at day 3, catching up with the control cell lines. Surprisingly, growth of the LeishIF4E-6(+/−) hemizygous mutant was reduced, and the cells did not reach a maximal concentration as control cells (Figure 8D). This reduction also correlates with the altered morphology of the LeishIF4E-6(+/−) mutant cells (Figure 7), as lack of motility could affect growth.

## 3. Discussion

We focus on understanding the function of the different cap-binding protein paralogs of *Leishmania* and their role in translation. The recent development of CRISPR-Cas9 technology assists us with unraveling the function of these translation factors. In this study, we have characterized the function of the least studied cap-binding protein paralog, LeishIF4E-6 in *Leishmania*.

Sequence analysis highlights that the LeishIF4E-6 amino acid sequence is different from other LeishIF4Es and its mammalian counterpart and shows the highest similarity with LeishIF4E-5 (43%). Its degree of conservation with the *T. brucei* ortholog is also limited, reaching only 56% similarity. The unique sequences of the *Leishmania* cap-binding proteins which are different from their mammalian counterparts could be the consequence of evolutionary divergence.

LeishIF4E-6 overexpressing cells show a significant reduction in their global translation as compared with cells that overexpress LeishIF4E-1-SBP. Unlike LeishIF4E-6, LeishIF4E-1 has been demonstrated to be involved in translation activity and is required for *Leishmania* growth and virulence [38]. The global translation was evaluated using the SUnSET assay that monitors the incorporation of puromycin into nascent chains and stops their elongation. Although we saw that episomal expression of transgenes caused a reduction in total translation possibly due to competition for translation factors [23,38,39], the reduction in translation in the LeishIF4E-6-SBP over-expression line was more significant as compared to controls. Therefore, global translation in the LeishIF4E-6 hemizygous knockout was less affected as compared to the LeishIF4E-6-SBP over-expressing line, although it was weaker than the Cas9/T7 control. The inhibitory effect of LeishIF4E-6 on translation activity and the lack of its m^7^GTP binding are unique to *Leishmania* and were not observed in the *T. brucei* ortholog, TbIF4E-6. LeishIF4E-6(+/−) showed ~50% reduction in its mRNA level when compared to WT control. The strong decrease in LeishIF4E-6 RNA could have affected the protein level of LeishIF4E-6, resulting in the effects observed on global translation and cell morphology. In the LeishIF4E-6 hemizygous mutants, we noticed a phenotypic defect in the mutant promastigotes which became small and round with reduced flagellar length. However, the morphological changes observed in the LeishIF4E-6(+/−) mutant cells correlate with the changes in cell motility of *T. brucei* cells, in which TbEIF4E-6 was silenced [42]. Our results showing that the hemizygous mutant cells growing slower could be due to the potentially essential function of LeishIF4E-6, but the growth defects could also be caused by the altered morphology of the hemizygous mutant. In contrast, overexpression of LeishIF4E-6 did not affect cell morphology, even though its global translation was reduced. Although these results were unexpected, they could be explained if LeishIF4E-6 functions in a transcript-specific manner.

Although LeishIF4E-6 is classified as a cap-binding protein and its predicted structure indicates that it has a typical cap-binding pocket, we did not observe any m^7^GTP-binding activities in any life cycle stage of *Leishmania*. However, we cannot exclude the possibility that LeishIF4E-6 has limited cap-binding activity, below the resolution of our affinity chromatography assays. In any case, its inability to bind m^7^GTP could suggest that LeishIF4E-6 may not be actively involved in translation initiation, supporting our observation of translation repression in LeishIF4E-6 overexpressing cells. The cap-binding pocket of the mammalian eIF4E consists of three tryptophan residues at positions 56, 102, and 166 of the mouse protein. These Trp residues are conserved in most eIF4Es but occasionally are replaced with other aromatic residues, such as Tyr or Phe. However, the substitution of conserved Trp residues may influence their binding of m^7^GTP cap, as observed for LeishIF4E-5, where the Trp residue that is analogous to the mouse Trp102 is replaced with Tyr [24]. Other residues may also assist the cap-binding activity, as basic residues within the pocket can interact with the acidic backbone of the cap structure. In the human eIF4E, Trp56 and Trp102 generate a π sandwich that stabilizes its binding to m^7^GTP. In LeishIF4E-6, these amino acids are substituted with Tyr36 and Phe82, respectively. Although these substitutions maintain aromatic residues in these positions, these changes can lead to a reduction of the π interactions between Phe82 and Tyr36 with the m^7^GTP, thus leading to a decreased affinity between LeishIFE-6 and m^7^GTP. Unlike our observation that LeishIF4E-6 does not bind m^7^GTP, the *T. brucei* ortholog, TbIF4E-6, was reported to associate with m^7^GTP. The different cap-binding activities observed for the *Leishmania* and *Trypanosoma* orthologs could originate from the altered residues that occupy the position which parallels Trp56 in the human eIF4E (Tyr in *Leishmania* and Phe in *T. brucei*).

Different eIF4E orthologs have been shown to form specific cap-binding complexes during the life cycle of *Leishmania* and *Trypanosoma*. We have identified the interacting proteins that are part of the LeishIF4E-6 complex using the pull-down analysis of tagged LeishIF4E-6. We noticed the enrichment of distinct classes of proteins including metabolic, transport-related, nuclear, mitochondrial, cytoskeletal, translation factors and chaperones that co-purified with LeishIF4E-6. Co-purification of chaperones was expected, as these are generally associated with overexpressed proteins. Among the translation factors, we observed a strong association of LeishIF4E-6 with LeishIF4G-5 and the G5-interacting protein (G5-IP). The LeishG5-IP contains the domains typically present in mRNA capping enzymes. Although the TbG5-IP was shown to be a homolog of the capping enzyme in *Crithidia fasciculata* and *T. brucei*, other proteins involved in the capping process were not present in the LeishIF4E-6 interactome [49].

We also noted that the canonical translation factor LeishIF4E-4 co-purified with LeishIF4E-6 rather efficiently. The co-purification of LeishIF4E-4 with LeishIF4E-6 could indicate that components of the LeishIF4E-6 complex were able to sequester LeishIF4E-4, the canonical translation factor, preventing its binding to the 5′ cap structure of mRNAs. This could be one of the possible mechanisms explaining the translation repression activity of LeishIF4E-6, although this possibility requires further investigation. We also noted that LeishIF4G-3 that usually accompanies LeishIF4E-4 was not found in the LeishIF4E-6 interactome.

Enrichment of the proteins associated with ribonucleoprotein granules could suggest that LeishIF4E-6 interacts with RNA granule proteins that are involved in translation repression and mRNA degradation. We observed that the ATP-dependent RNA helicase DHH1 (LmjF.35.0370) was enriched in the LeishIF4E-6 associated proteins. DHH1 is known to regulate the balance between active translation, P-bodies formation and cytoplasmic decay of mRNAs [50]. However, in trypanosomes, it is also known to play a selective role in determining the expression levels of developmentally regulated mRNAs [51]. Furthermore, a *Leishmania* homolog (LmjF.22.1600) of TbALPH1 was also found to be enriched in the LeishIF4E-6 interactome (Table S1). In Trypanosomes, TbALPH1 is an mRNA decapping enzyme, identified in stress granules, and it also co-localizes with XRNA (5′-3′ exoribonuclease) in the posterior pole granules [48]. Furthermore, we found that PUF1 (LmjF.36.0050), the Alba-domain protein 1 (LmjF.13.0450) and an RNA binding protein (LmjF.18.0590) co-purified with LeishIF4E-6. In *Leishmania* and *T. brucei*, PUF1 was reported to localize to cytoplasmic starvation-induced stress granules, which store mRNAs following their stalled translation [36,52]. In higher eukaryotes, PUF proteins are involved in translation repression either by blocking cap-binding events or by recruiting the deadenylating complex to remove the poly (A) tails [53]. In trypanosomatids, the association of PUF1 with stress granules suggests that it could have a role in translation repression.

The *L. infantum* homolog of the Alba-domain protein 1 (LiAlba1, LmjF.13.0450) is LINF_130009400 (previously Linj.13.0270). LiAlba1 and LiAlba3 were reported to form a complex with other RNA-binding proteins, ribosomal subunits, and translation factors and both proteins migrate to heavier sucrose fractions along with ribosomal proteins upon conditions inducing translational decline. This suggests a potential role of LiAlba1 in translational repression [54]. The *L. infantum* homolog of the RNA binding protein (LmjF.180590) LINF_180010900 (previously Linj.18.0590) was reported to interact with LiAlba1 only during the promastigote stage. LINF_180010900 is a 44 kDa RNA binding protein specific to *Leishmania* [54]. The association of PUF1 with LeishIF4E-6 shows that the latter could have a role in post-transcriptional RNA turnover [55]. Enrichment of metabolic enzymes in the LeishIF4E-6 interactome could result from their moon-lighting RNA binding activity [47].

The GO enrichment analysis highlighted the co-purification of proteins of the tethering complex group that have a primary function in the trafficking of proteins. These included orthologs of the conserved oligomeric complex 6, COG6 (LmjF.34.0370), that was proposed to function in membrane tethering during vesicular trafficking at the Golgi apparatus [56]. Another protein, the exocyst complex component Exo99 (LmjF.21.1569) is an essential subunit of the nonameric exocyst complex first identified in *Trypanosoma brucei* [57]. In trypanosomes, Exo99 has a role in viability, morphology and trafficking as well as in maintaining the surface proteome. A TRAPPC-like protein (LmjF.16.1400) co-purifies with LeishIF4E-6. The subunit-like protein TRAPPC which in mammals is part of the trafficking protein particle complex (TRAPP complex), which mediates the contact between vesicles and target membranes [58]. The GO enrichment analysis also identified components of the contractile vacuoles with LeishIF4E-6, based on the presence of calmodulin in the interactome. Contractile vacuoles are involved in the regulation of cell volume, by control of the water quantity within the cell and are involved in adaptation to high osmolarity conditions. The involvement of calmodulin in contractile vacuoles has been reported [58], and calmodulin, which is enriched in the LeishIF4E-6 interactome, is rich in the membrane of contractile vacuole complexes. Calmodulin is a cytosolic calcium-binding messenger protein that mediates Ca2+ regulation in a wide range of cellular processes, such as activation of regulatory enzymes (e.g., adenylyl cyclases, ATPase) [59] and reversible modulation of calcium-dependent processes [60,61]. Given the above, co-purification of contractile vacuole components with LeishIF4E-6 could possibly indicate why cell morphology is altered in the LeishIF4E-6(+/−) mutant [62,63], although other mechanisms for this effect cannot be ruled out.

We identified LeishIF4G-5 as a binding partner of LeishIF4E-6 through mass spectrometry analysis of proteins that were pulled down with LeishIF4E-6. The association between LeishIF4G-5 and LeishIF4E-6 was verified in vitro, by monitoring the interaction between LeishIF4E-6 and recombinant LeishIF4G-5. The interaction between the two proteins was also observed in *T. brucei*, suggesting that they form an evolutionarily conserved complex. The YXXXXLϕ domain that is found in eIF4E-binding proteins is also shared by the 4E-interacting proteins in *Leishmania* [21,39]. Sequence analysis of LeishIF4G-5 identified two conserved putative YXXXXLϕ domains, one at the center of the protein and another at the C-terminus of LeishIF4G-5. However, the latter element has a cysteine residue at its 6th position of the suggested 4E-binding domain. It should be noted though that analysis of the interaction between LeishIF4G-3 and LeishIF4E-4 showed that the YXXXXLϕ domain in LeishIF4G-3 was only partially conserved, as its 6th position is occupied by Aspartic acid, rather than by a Lysine residue [20]. Thus, the suggested 4E-binding domain at that C-terminus of LeishIF4G-5 could be functional. The presence of two 4E-binding motifs in LeishIF4G-5 could also suggest that this protein is capable of binding to both LeishIF4E-6 and LeishIF4E-4. The co-purification of LeishIF4E-4 with LeishIF4E-6 could be mediated by other specific proteins in the LeishIF4E-6 complex, which were not yet identified. The presence of LeishIF4E-4 in this complex could therefore potentially bring LeishIF4E-4 bound transcripts to interact with the LeishIF4E-6 bound proteins that are involved in RNA inactivation such as TbALPH1 homolog, PUF1 and DHH1. Further investigations are required to establish the interaction between LeishIF4G-5 and LeishIF4E-4. We also observed that LeishIF4G-5 is subjected to proteolysis. To confirm that the cleavage products were derived from LeishIF4G-5, we carried out the mass spectrometry of each band detected in Western blots and found LeishIF4G-5 specific peptides. The biological role of this cleavage is still not clear.

Overall, we report that LeishIF4E-6 is a unique protein that does not associate with the m^7^GTP cap. Its inability to interact with the mRNA cap suggests that LeishIF4E-6 could not promote translation initiation, possibly suggesting that LeishIF4E-6 could have a role in translation repression, as suggested by the global translation assays. The interactome of LeishIF4E-6 is rich with proteins involved in RNA inactivation such as DHH1, the TbALPH1 homolog, PUF1 and the Alba-domain protein 1. These proteins have an established role in translation repression through mRNA degradation (ALPH1 homolog) or via sequestering of mRNAs to dedicated storage granules (DHH1, PUF1). Although LeishIF4E-6 does not directly interact with the LeishIF4E-4, its interaction with LeishIF4G-5 could tether LeishIF4E-4 to the LeishIF4E-6 complex. Such an interaction could potentially sequester LeishIF4E-4 to dedicated foci that contain enzymes that have an overall inhibitory effect on translation, such as DHH1, PUF1, and the Alba-domain protein 1. Moreover, the presence of the decapping enzyme homolog of TbALPH1 in the complex could further support the inhibitory nature of LeishIF4E-6 on translation. This could be a potential mechanism by which LeishIF4E-6 regulates protein translation. The requirement for the LeishIF4E-6 and LeishIF4G-5 complex formation presents a unique and intriguing regulatory mechanism that will be further investigated.

## 4. Materials and Methods

### 4.1. Organisms and Cell Culture

*Leishmania amazonensis* (MHOM LTB0016) and *Leishmania mexicana* (M379) promastigotes were cultured at 25 °C in Medium 199 [M199, (pH 7.4)] (Biological Industries, Beit Haemek, Israel) containing 10% fetal calf serum (FCS, Biological Industries, Beit Haemek, Israel), 5 µg/mL hemin, 0.1 mM adenine (Sigma, St. Louis, MO, USA), 40 mM HEPES (Sigma, St. Louis, MO, USA), 4 mM L-glutamine (Biological Industries, Beit Haemek, Israel), 100 U/mL penicillin and 100 µg/mL and streptomycin (Biological Industries, Beit Haemek, Israel). *Leishmania amazonensis* axenic amastigotes were generated using promastigote cells from late log phase (3.6 × 107 cells/mL) which were washed twice with phosphate-buffered saline (PBS) and resuspended in M199, containing 25% FCS, 5 µg/mL hemin, 0.1 mM adenine, 40 mM HEPES pH 5.5 (adjusted by using 0.5 M succinic acid), 4 mM L-glutamine, 100 U/mL penicillin and 100 µg/mL streptomycin. After resuspension in axenic amastigote specific media, the cells were further grown at 33° C for four days with constant shaking. 

### 4.2. Generation of Transgenic Leishmania Overexpressing the SBP Tagged LeishIF4E-6

The LeishIF4E-6 open reading frame (ORF, 549 bp) was amplified from *L. amazonensis* genomic DNA using gene-specific primers, and cloned into a pX-derived transfection cassette, pX-H-target ORF-H-SBP between the BamHI/XbaI sites. In this transfection, cassette H represents the intergenic region of HSP83 derived from the genomic locus [21] of *Leishmania* and SBP (streptavidin-binding peptide, 6 kDa) is the C-terminal tag. Mid-log phase *L. amazonensis* cells (50 mL) were transfected with 40 μg of the construct, pX-H-LeishIF4E-6-SBP-H- following a published protocol [64]. The positive clones were selected for resistance to G418 (200 μg/mL).

### 4.3. Sequence Analysis of LeishIF4E-6

A multiple sequence alignment was carried out and the secondary structure of LeishIF4E-6 was predicted. The ORFs of LeishIF4E-6 from various *Leishmania* and Trypanosoma species along with mammalian and *L. amazonensis* LeishIF4E-1 were aligned using the Jalview (2.11.1.4) alignment tool [65]. The sequences used were *Mus musculus* eIF4E-1 (ENSMUSG00000028156), *L. amazonensis* LeishIF4E-1 (LAMA_000544700), *L. amazonensis* LeishIF4E-6 (LAMA_000504800); *L. mexicana* LeishIF4E-6 (LmxM.26.0240) *L. donovani* LeishIF4E-6 (LdBPK.26.2.000230), and *T. brucei* TbIF4E-6 (Tb927.7.1670). The alignment file that was generated with Jalview was first saved in FASTA format. Then, the PDB file (6YLT, doi:10.2210/pdb6YLT/pdb) of *Mus musculus* eIF4E-1 was downloaded from the protein database (https://www.rcsb.org/structure/6YLT, accessed on 5 July 2021). The final sequence and structural alignment showing the predicted secondary structure was developed using the downloaded PDB file (6YLT, doi:10.2210/pdb6YLT/pdb) of *Mus musculus* eIF4E-1 along with the FASTA alignment file, using the online ESPript 3 tool ESPript 3 (ibcp.fr). The separate alignment *L. amazonensis* of LeishIF4E-6 and *T. brucei* TbIF4E-6 were also carried out using Jalview 2.11.1.4, to highlight the homology between these two sequences. Sequence similarities of LeishIF4E-6 with various LeishIF4Es from *Leishmania*, *Mus musculus* eIF4E1 and *Trypanosoma* TbIF4E-6 were generated by the EMBOSS needle tool (https://www.ebi.ac.uk/Tools/psa/emboss_needle/, accessed on 6 May 2021). Similarly, sequence similarities of LeishIF4G-5 with various LeishIF4Gs from *Leishmania* and *Mus musculus* eIF4G were also generated.

### 4.4. Structural Homology Modeling of LeishIF4E-6

Structural homology modeling was performed on LeishIF4E-6 via SWISS-MODEL workspace [65]. Several modeling structures were chosen and superposed on their known template structures using WinCoot 0.9.4.1 [66] and the best template structure was chosen based on the quality of the solved structures, the presence of ligand, and the completeness and the orientation of the pocket loops. Images were prepared via ChimeraX 1.2.4 and GIMP 2.10.24 used for the graphical editing.

### 4.5. Confocal Microscopy to Monitor the Localization of LeishIF4E-6-SBP

*L. amazonensis* promastigotes overexpressing LeishIF4E-6-SBP were examined by confocal microscopy. The transgenic cells were cultured in 8 well μ-Slide plates (Ibidi GmbH, Gräfelfing, Germany) which fits in the microscope chamber, and mid-log cells (~107 cells/mL) were processed for imaging. The cells were washed once in serum-free M199 media and then fixed in 2% paraformaldehyde in PBS for 20 min at room temperature and then washed with PBS once. Cells were permeabilized with 0.1% Triton X-100 in PBS, washed again with PBS and blocked with 2% bovine serum albumin (BSA) in PBS at room temperature for 1 h. The fixed cells were then incubated with a monoclonal antibody against the SBP tag (1:100) (Millipore, Ternecula, CA, USA) for an hour at room temperature. The cells were washed again with PBS and incubated with and a secondary anti-mouse IgG DyLight 488 (3:500) (KPL, Milford, CT, USA) for an hour at room temperature. The nuclear and kinetoplast DNA was stained using 4′, 6-diamidino-2-phenylindole (DAPI; 1 μg/mL of PBS; Sigma). An inverted Zeiss LSM 880 Axio-observer Z1 confocal laser-scanning microscope with Airyscan detector was used to capture the images. A Plan-Apochromat 63x/1.4 oil, 1.4 Numeric aperture (NA), differential Interference Contrast (DIC) objective was used to visualize the cells. Images were recorded at a 512 x 512 pixels format along with 8x digital zoom for an enlarged view of *Leishmania* promastigote. Airyscan processing of the images was carried out using Zen lite software (Carl Zeiss AG, Oberkochen, Germany). ImageJ software was further used to generate a single representative section of Z-projection with maximum intensity [67].

### 4.6. Monitoring Global Translation Activity

Global translation in transgenic *L. amazonensis* promastigotes overexpressing LeishIF4E-6-SBP along with control wild type (WT) cells and cells overexpressing the transgenic LeishIF4E-1-SBP and the chloramphenicol acetyltransferase (CAT) reporter protein (flanked by the HSP83 intergenic regions (i), pX-iCATi, was monitored using the SUrface SEnsing of Translation (SUnSET) assay. This assay is based on the incorporation of puromycin, a structural tRNA analog, into the growing polypeptide in the A site of the translating ribosome [68]. Quantification of puromycin incorporated serves as an indication for global translation in the examined cells. The different cell lines described above were incubated with 1 µg/mL of puromycin (Sigma, St. Louis, MO, USA) for 30 min, washed twice with PBS and once with PRS^+^ buffer (PRS: 35 mM HEPES, 100 mM KCl, 10 mM MgCl_2_,1 mM dithiothreitol, DTT; PRS^+^: PRS supplemented with Phosphatase inhibitors [20 mM NaF, 50 mM β-glycerophosphatase] and Protease inhibitors [2× protease inhibitors cocktail, 2 mM iodoacetamide]). The washed cell pellets were resuspended in 300 µL of PRS^+^ buffer and Laemmli sample buffer (2×) and boiled for 5 min. Cells treated with Cycloheximide (100 μg/mL) (Sigma, St. Louis, MO, USA) prior to the addition of puromycin served as the negative control for translation. The samples were resolved over 10% SDS- SDS-polyacrylamide (PAGE) gels. Western analysis was carried out using specific primary monoclonal antibodies against puromycin (DSHB, University of Iowa, Iowa, USA; 1:1000) and secondary peroxidase-labeled anti-mouse antibodies (KPL, Milford, CT, USA; 1:10,000)

### 4.7. Affinity Purification over m^7^GTP-Agarose

Transgenic lines overexpressing the SBP-tagged LeishIF4E-6 and LeishIF4E-1 (as a positive control) were used in the assay. The cells (∼10^9^) were washed twice with PBS and once with column buffer, (CB, 20 mM HEPES, pH 7.4, 2 mM EDTA, 1 mM DTT and 50 mM NaCl). The cell pellets were resuspended in 1.2 mL of CB^+^ which consisted of CB containing protease and phosphatase inhibitors (a commercial mix of protease inhibitors (Sigma), 4 mM iodoacetamide, 25 mM sodium fluoride and 55 mM β-glycerophosphate). *Leishmania* cells were lysed with 1% Triton X-100 in CB^+^ on ice for 5 min. The supernatants were clarified by centrifugation at 20,000× *g* for 20 min at 4 °C. The clarified supernatants were then incubated for 2 h with m^7^GTP -agarose resin (75 μL) (Jena Biosciences, Jena, Germany) that were pre-equilibrated with CB. The m^7^GTP -agarose resins bound to protein complexes were washed with CB twice and once with CB supplemented with 100 μM GTP. Finally, the elution of the cap-binding complexes was carried out with CB^+^ containing 200 μM of free m^7^GTP. The eluted fractions were precipitated with trichloroacetic acid (TCA), at a final concentration of 10% at 4 °C overnight under constant shaking. The proteins were spun down at 20,000× *g* for 20 min at 4 °C and the pellets were washed with 100% chilled acetone, briefly dried and resuspended in Laemmli sample buffer. Aliquots derived from the supernatant (5%), the flow-through (5%), wash (50%) and elution (50%) fractions were resolved over 10% SDS-PAGE gels, blotted and western analysis was carried out with specific monoclonal antibodies against SBP tag.

### 4.8. Affinity Purification over Streptavidin-Sepharose Beads

Lysates of cells overexpressing SBP-tagged LeishIF4E-6 and Luciferase were affinity-purified over streptavidin-Sepharose beads. The cells (~109) were washed twice with PBS, once with PRS, then lysed with 1% Triton X-100 in 1.2 mL PRS^+^ for 5 min in ice. The cell lysates were clarified by centrifugation at 20,000× *g* for 20 min at 4 °C. The clarified supernatants (1.2 mL each) were then incubated for 2 h with 75 μL streptavidin-Sepharose beads (GE Healthcare, Buckinghamshire, UK) pre-equilibrated with PRS buffer for 2 h. After collecting the flow-through, the beads were washed three times with PRS^+^ containing 0.1% NP-40. The final elution was carried out with 5 mM biotin in PRS^+^ supplemented with 0.1% NP-40. The eluted fractions were precipitated with trichloroacetic acid (TCA) at a final concentration of 10% at 4 °C overnight with constant shaking. The proteins were precipitated by centrifugation at 20,000× *g* for 20 min at 4 °C washed with 100% chilled acetone and finally resuspended in Laemmli sample buffer. Aliquots of the different fractions including the supernatant (5%), the flow-through (5%), the final wash (50%) and the elution (50%) were separated over 12% SDS-PAGE gels, blotted and western analysis was carried out with specific antibodies. The eluted samples from the SBP-tagged LeishIF4E-6 and Luciferase cell lines were then subjected to mass spectrometry analysis.

### 4.9. Proteomic Analysis by Mass Spectrometry

Lysates of cells overexpressing SBP-tagged LeishIF4E-6 and Luciferase were affinity-purified over streptavidin-Sepharose beads. The eluted proteins were precipitated with 10% TCA, and the pellets were washed with 100% chilled acetone. The protein pellets were resuspended in Laemmali’s buffer and further separated over 12% SDS-PAGE gels that were stained with Coomassie Brilliant Blue. The relevant lanes were excised and subjected to mass spectrometry analysis in the Smoler Proteomics Center at the Technion, Israel.

Mass Spectrometry-Proteins were reduced by adding 3 mM DTT at 60 °C for 30 min. This was followed by modification with 10 mM iodoacetamide in 100 mM ammonium bicarbonate (Promega, Madison, WI, USA) (30 min at room temperature). The proteins were then digested with trypsin (Promega) in 10 mM ammonium bicarbonate at 37 °C overnight. The trypsin-digested peptides were desalted, dried, and resuspended in 0.1% formic acid and finally resolved by the reverse phase chromatography over a 30 min linear gradient of 5% to 35% acetonitrile and 0.1% formic acid in water, a 15 min gradient of 35% to 95% acetonitrile and 0.1% formic acid in water and a 15 min gradient of 95% acetonitrile and 0.1% formic acid in water at a flow rate of 0.15 µL/min. Mass spectrometry (MS) was carried out using a Q Exactive Plus mass spectrometer (Thermo Fischer Scientific, Waltham, MA, USA) in the positive mode set to conduct a repetitively full MS scan followed by the high energy collision dissociation of the 10 dominant ions selected from the first MS scan. A mass tolerance of 10 ppm for precursor masses and 20 ppm for fragment ions was set.

Statistical Analysis—MaxQuant software (version 1.5.2.8) [69] was used to analyze the raw data generated by mass spectrometry to identify the proteins that were identified in the lysates of cells overexpressing LeishIF4E-6 as compared to the luciferase-SBP control The raw data were searched against the highly annotated L. major Friedlin strain proteins enlisted in the TriTrypDB database [46]. Protein identification parameters were set at less than a 1% false discovery rate. The MaxQuant parameter settings included a minimum of 1 razor/unique peptide for identification, a minimum peptide length of six amino acids and a maximum of two mis-cleavages. Summed peptide intensities were used for the protein quantification. Missing intensities of the proteins in control Luciferase samples were substituted with values close to the baseline. To identify the proteins that were enriched in the LeishIF4E-6 interactome, the log_2_ of iBAQ intensities [70] were compared between three LeishIF4E-6-SBP biological repeats and three luciferase- SBP repeats on the Perseus software platform [71], using the *t*-test. Adjusted *p*-values were corrected using permutation-based false discovery rate (FDR) = 0.05 and the number of randomizations = 250. These are marked as Padj [71]. The enrichment threshold was set to a log_2_ fold change > 1.6 and a *p*-value < 0.05. The annotated proteins were first categorized manually based on the functions. Proteins enriched in the LeishIF4E-6 as compared to Luciferase-SBP were manually categorized into different groups based on their functions and represented by the pie chart.

### 4.10. Gene Ontology (GO) Annotation of Enriched Proteins

The proteins that were enriched in the LeishIF4E-6-SBP interactome as compared to the control Luciferase-SBP were further annotated by the GO Annotation tool in TriTrypDB, based on the cellular components’ parameter. The threshold for this analysis of proteins based on their GO terms was set at four-fold, with a *p*-value < 0.01. This threshold eliminated most of the general groups that represented parental GO terms. GO terms based on only a single protein were also filtered out.

### 4.11. Cloning and Expression of Recombinant GST-Tagged LeishIF4E-6

LeishIF4E-6 (549 bp) ORF was amplified by PCR using *L. amazonensis* genomic DNA with the specific set of forward primer 5′-gctgaattcATGGCCGATAGTAATCCCACCA-3′ and reverse primer 5′-gctctagaCTA CTT GAA GGG GCG AGC GCT-3′. Lower case letters represent restriction sites EcoRI in forward primer and XbaI in reverse primer while upper case letters are the sequences from LeishIF4E-6 ORF. The pGST-parallel expression plasmid was used for cloning of LeishIF4E-6 with an N-terminus GST tag [72]. BL21 *E. coli* cells were transformed with the plasmid clone containing GST-tagged LeishIF4E-6. The BL21 *E. coli* cells were grown at 37 °C until OD_600_ = 0.5, when expression was induced by the addition of 0.5 mM Isopropyl-β-d-1-thiogalactopyranoside (IPTG). *E. coli* cells were further incubated for 14–16 h at 18 °C after IPTG addition.

### 4.12. Interaction between Recombinant GST-LeishIF4E-6 and LeishIF4G-5-SBP

*Transgenic L. amazonensis* cells overexpressing the SBP-tagged LeishIF4G-5 and BL21 *E. coli* cells expressing the recombinant GST-tagged LeishIF4E-6 were used for the interaction assay. Mid log phase *Leishmania* cells (~8× 10^6^ cells/m) overexpressing the SBP tagged LeishIF4G-5 were washed with PBS twice and once with disruption buffer (DB, 20 mM Tris-HCl pH 8.0, 200 mM NaCl, 1 mM EDTA, 5 mM MgCl2 and 5% glycerol). The cell pellets were then resuspended in the same buffer (DB) containing a cocktail of protease inhibitors (Sigma) and 2 mM iodoacetamide, along with phosphatase inhibitors (20 mM sodium fluoride, 50 mM β-glycerophosphatase) and 0.1% NP-40 (DB^+^). Cell lysis was carried out with 1% Triton X-100 for 5 min on ice. Clarified supernatant was further obtained by subjecting the lysates to centrifugation at 20,000× *g* for 20 min at 4 °C. Clarified supernatant containing SBP-tagged LeishIF4G-5 was then incubated with pre-equilibrated (with DB^+^ buffer) streptavidin-Sepharose beads (75 μL, GE Healthcare) for 2 h at 4 °C with constant shaking. The beads were then washed three times with DB^+^. Simultaneously, IPTG induced BL21 *E. coli* cells (200 mL) expressing the GST-tagged LeishIF4E-6 were washed twice with PBS, and once with DB. The BL21 *E. coli* cell pellets were resuspended in DB^+^ and disrupted in a French Press apparatus at 1500 twice. The disrupted cells were centrifuged at 45,000× *g* rpm in a Ti70 rotor (Beckman Coulter) for 45 min. The clarified bacterial supernatant containing the GST-tagged LeishIF4E-6 (10 mL) was then incubated with the streptavidin-Sepharose beads that were pre-bound with the SBP tagged LeishIF4G-5. This incubation was set for 2 h at 4 °C with constant shaking. After this incubation, the flow-through fraction was collected and the beads were washed five times with 1 mL of DB^+^. The final elution was carried out using 5 mM of biotin in 1 mL DB^+^. Aliquots derived from the supernatant (S, 2%), flow-through (FT, 2%), wash (W, 25%) and elution (E, 25%) fractions were separated over 12% SDS-PAGE and subjected to western analysis using antibodies against the SBP tag to detect LeishIF4G-5 (Millipore, Temecula, CA, USA). Antibodies against the GST tag (Invitrogen 1:1000) were used to detect LeishIF4E-6. 

### 4.13. CRISPR-Cas9-Mediated Generation of a Hemizygous LeishIF4E-6 Mutant

The different plasmids optimized for the CRISPR-Cas9 mediated gene editing in *Leishmania* were a kind gift from Eva Gluenz (University of Oxford, Oxford, UK) [73]. The pTB007 plasmid was used to generate a *Leishmania mexicana* cell line that expressed Streptococcus pyogenes CRISPR-associated protein 9 endonuclease and the T7 RNA polymerase gene (Cas9/T7). This cell line was selected for hygromycin resistance (200 μg/mL).

A LeishIF4E-6 hemizygous mutant was generated using different PCR-amplified products. The two 5′ and 3′ sgRNAs were derived from the respective untranslated region of LeishIF4E-6, to target the double-strand breaks. Other PCR products included the two different repair cassette fragments that introduced either the G418 or the blasticidin marker into the double-strand breaks in the genome. All four PCR products were electroporated into mid-log-phase transgenic cells overexpressing Cas9 and the T7 RNA polymerase. Cells were further selected for resistance to 200 μg/mL G418 [38,73], or 20 μg/mL Blasticidin or both. The sgRNA sequences that were used to delete the LeishIF4E-6 gene were obtained from http://leishgedit.net (5 July 2020).

PCR Amplification of sgRNA Templates. LeishIF4E-6 specific 5′ and 3′ guide RNAs fragments for cleavage upstream and downstream of the LeishIF4E-6 target gene were generated by PCR. The template for this PCR included two partially complementary long oligonucleotides; the common sgRNA scaffold oligonucleotide (5′-AAAGCACCGACTCGGTGCCACTTTTTCAAGTTGATAACGGACTAGCCTTATTTTAACTTGCTATTTCTAGCTCTAAAAC-3′), and another oligonucleotide that included the T7 RNA polymerase promoter (lowercase letters at the beginning of the sequence) fused to the gRNA (5′ or 3′) targeting LeishIF4E-6 (capital letters) and a short sequence overlapping the scaffold fragment (lowercase letters at the end of the sequence). The two individual template oligonucleotides designed for targeting the double-strand breaks at the 5′ and 3′ ends of LeishIF4E-6 were 5′-gaaattaatacgactcactataggCCGACTCGTCTGCGTAGCCGgttttagagctagaaatagc-3′ and 5′-gaaattaatacgactcactataggTGCGCATATGTTCATCAGTGgttttagagctagaaatagc-3′, respectively. These two template fragments (1 μg each) were annealed to their partially complementary sequence and further amplified with a pair of small primers (2 μM each) derived from the T7 promoter (G00F, 5′-TTAATACGACTCACTATAGG-3′) and the common scaffold fragment (G00R, 5′-GCACCGACTCGGTGCCACTT-3′). PCR conditions were described in [38]. All PCR products were gel extracted and sterilized at 94 °C for 5 min before transfection.

PCR Amplification of the LeishIF4E-6 Replacement Fragment. The LeishIF4E-6 replacement fragment was amplified by PCR using the pTNeo plasmid as a template. The primers were (5′-CGCCACCGTTCCGCCGGCTTCCTTCAGCCTgtataatgcagacctgctgc-3′ [forward]) and 5′-ACACTCTATACACACATGCGCACACACGTAccaatttgagagacctgtgc-3′ [reverse]). The capital letters represent 5′ and 3′ UTR sequences in the Forward and Reverse primers, respectively. The lowercase letters represent the region on the pTNeo plasmid that flanks the UTR adjacent to the antibiotic resistance gene (G418 or Blasticidin). These primer pairs were designed based on the LeishGEdit database (http://www.leishgedit.net/Home.html). The LeishIF4E-6 replacement fragment consisted of G418 sequences flanked by LeishIF4E-6 specific UTRs that promote the integration of the drug resistance marker by homologous recombination at the target site. PCR conditions were described in [38]. All PCR products were gel extracted and heated at 94 °C for 5 min before electroporation in *Leishmania* cells.

Diagnostic PCR to Confirm the Deletion of LeishIF4E-6. Genomic DNA was isolated from the *Leishmania* cells transfected with different PCR products to knock out the LeishIF4E-6 gene after 14 days of G418 drug selection by using DNeasy Blood & Tissue Kit (Qiagen, Germantown, MA, USA). We carried out several diagnostic PCRs to detect the deletion of the LeishIF4E-6 gene, using different primer pairs. The first PCR included primers derived from LeishIF4E-6 UTRs, a forward primer from the 5′UTR (P1: 5′-CACAGCATCTCCATTCCTTCTT-3′) and a reverse primer derived from the 3′ UTR of LeishIF4E-6 (P2: 5′-TCTCCACCACCACGGAAT-3′). Another PCR reaction was performed to analyze the insertion of the G418 resistance gene using the G418 ORF primers which were G418 Forward (P3: 5′-GCCCGGTTCTTTTTGTCAAGAC-3′) and G418 Reverse (P4: 5′-GTCACGACGAGATCATCATCGCCG-3′). Another PCR reaction was carried out to confirm the insertion of the G418 cassette at the target site. This PCR was performed with primers derived from the G418 ORF (Forward P3) and the LeishIF4E-6 3′UTR (Reverse P2). Genomic DNA from the positive control Cas9/T7 overexpressing *L*. *mexicana* cells was used to detect the presence of the LeishIF4E-6 gene using the primer set derived from the 5′ and 3′ UTRs. The PCR conditions were described in [38].

### 4.14. Quantitative Real-Time PCR

Total RNA was isolated from *L. amazonensis* promastigotes overexpressing LeishIF4E-6-SBP, along with control wild-type (WT) cells and cells overexpressing the transgenic LeishIF4E-1-SBP and the chloramphenicol acetyltransferase (CAT) reporter protein. Total RNA was also extracted from *L. mexicana* promastigotes overexpressing Cas9/T7, along with control wild type (WT) cells and *L. mexicana* 4E6+/− hemizygous mutant. The RNA was extracted from day 2 cells using the TRI reagent (Sigma), following the manufacturer’s instructions. The purified RNA was subjected to DNAse treatment by incubation at room temperature for 20 min followed by the heat inactivation of the DNAse (Thermo Fisher Scientific, Bleiswijk, Netherlands) at 65 °C for 10 min. Finally, the RNA samples were stored at −80 °C. 

cDNA was synthesized from 1 μg total RNA from different cell lines in a 20 μL reaction mixture by the use of High-Capacity cDNA Reverse Transcription kit (Applied Biosystems, Foster City, CA, USA) according to the manufacturer’s instructions. The cDNA was diluted 1:5 and 1 µL was used for the RT-PCR reaction. Reverse transcription reaction conditions included 25 °C for 10 min, 37 °C for 120 min, and 85 °C for 5 min followed by cooling at 4 °C. The real-time PCR reactions were carried out using a Applied Biosystem (cat # 4376600, StepOnePlus Real-Time PCR System) platform using the Fast SYBR Green Master Mix (Applied Biosystems, Foster City, CA, USA). Reaction mixtures included 5 μL 2X Fast SYBR Green Master Mix, 1 μL of each forward and reverse primers (5 pm each), 2 μL nuclease-free H_2_O and 1 μL cDNA. The primer sequences used for LeishIF4E-6 genes were FP (GAACGACGACAACCTCGTCT) and RP (TTGCAGGCGTCACGACTAAT). The primer sequences used for the endogenous control gene GAPDH were FP (GGGTAAGCTCGGTGTGGATTAC) and RP (CTGGTTCACACCCATCACGA). The PCR conditions were: 95 °C for 20 sec for initial activation followed by 40 cycles of 95 °C for 3 sec, and 60 °C for 30 sec. The PCR reaction was followed by a melting curve analysis to check the purity of the product. This was done at 95 °C for 15 sec, 60 °C for 60 sec and 95 °C for 15 sec. QRT-PCR reactions were analyzed using StepOne™ Software provided with the instrument. The relative expression of the genes to check fold differences was calculated by using the 2^−ΔΔC^_T_ formula where endogenous control GAPDH was used as the normalizer. The values of three replicates were represented as a dot plot graph. The statistical analysis was carried out using GraphPad Prism 5 and significant differences were estimated by non-parametric T-test using a paired *t*-test.

## Data Availability

All data are provided as part of this manuscript, in the main figures and in the supplemental materials.

## Supplementary Materials

The following are available online at https://www.mdpi.com/article/10.3390/ijms222312720/s1.
