# Supplementary material for: Characterization of an Atypical eIF4E Ortholog in Leishmania, LeishIF4E-6
Source: Int J Mol Sci. 2021 Nov 24;22(23):12720. doi: 10.3390/ijms222312720 (PMC8657474; doi:10.3390/ijms222312720)
Supplement: Supplementary file 1 [file ijms-22-12720-s001.zip › ijms-1437572-supplementary/Supplementary figures.pdf]

A

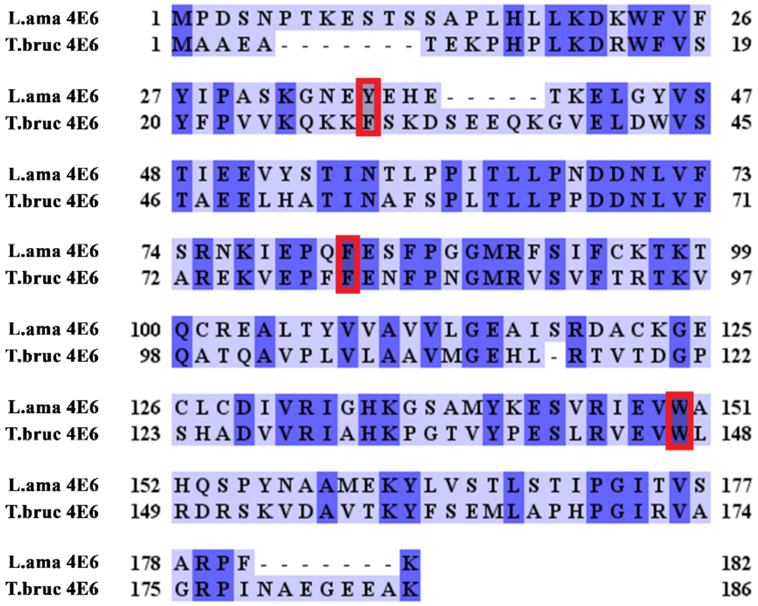

B

4Es	% Similarity to *L. amazonensis* 4E6
M. musculus eIF4E1	31.4
L. amazonensis 4E1	27.3
L. amazonensis 4E2	18.7
L. amazonensis 4E3	21.4
L. amazonensis 4E4	24.1
L. amazonensis 4E5	43.2
T. brucei 4E6	56.2

Figure S1. (A) Alignment of *L. amazonensis* eIF4E-6 with *T. brucei* counterpart. The eIF4E-6 sequences from *L. amazonensis* and *T. brucei were* subjected to sequence alignment using Jalview 2.11.1.4. Dashes represent spaces that were inserted to allow better alignment. Conserved Tryptophan, Tyrosine and Phenylalanine residues involved in the cap-binding activity are highlighte in red boxes. (B) **Sequence similarity of *L. amazonensis* LeishIF4E-6, with various other paralogs and mammalian eIF4E-1.** The table shows percent similarities between the LeishIF4E-6 and different *Leishmania* LeishIF4Es, the *Mus Musculus* eIF4E and *T. brucei* TbIF4E-6. Percent similarities were generated by EMBOSS needle (https://www.ebi.ac.uk/Tools/psa/emboss_needle/).

A

```
>LAMA_000167100.1 LeishIF4G5 780 amino acids
MNKNYPGRITSAPLAQQARGMSLRPGAPASFLDRAQPAGAGSGISAQQPHHHSSLPRAPRAK
PPTQLQTPAPAPPPPKKEAEEECSGAAEVVPSEVSYPEDHIYNVEDFIKLRKCQAPVPSEVM
EYARAMWKEMPENMDDMENNSLRDNLFREQNASTMSKVMTDRKINNEVLGILGKVTASNLEK
MKKELTDLPIRQSTKEEIDEVIKVFFNKSTKPEDSCYTHLYVQLIAHLISSIGEREEAGRMI
RNEVLRQCRSTFMNSGAEAVELEKRMATMSPEDAEMERIQFSGKQKANIQFLGLMFTSRLVR
QKVVHAVLDSLLYGPGHRRHIPTDYSLIHFMELLQVCGPHLDAAFYEDPLPRYRETITELSN
SHPQKRIQFLLQNFLETMNNNWVPLHGPGARRTEGGGNAAQGNNNSSGTLLNNNGRGGTLPT
MPPAPMPIQEAQPRIPDYEEFSKVMDDFFMSSSVDEIVSVMSSIPPEVVVVYCTKWLGRYIN
TYKYTAERTRLGELFETLMKKGALTTEQAQEALLQHLQKSAEEELFTDIPKYFVHWASLIKH
GHSVFPYSLHTKVLNMLVDNHVSIEVIANMVRDVEADTKPDQLKDLKPQDRFRVLQALLRYT
PPMFVHESDDDQQPKQTLLDLVGAIDPEVTYFNELCTSYDDDNFHASPALSDMQKQSPYLCA
SAFFTFVRYDVNFLCTQYKELLRKIFTARPADTLLVEVYLQWRCLGCSHRFLFAFIRKVLDV
VNNRADVLNKLSTQLRTTFKEDTLVSLMEETIKERK
```

B

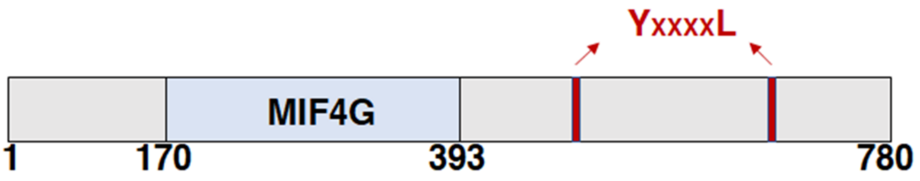

C

4Gs	% Similarity to *L. amazonensis* 4G5
M. musculus eIF4G1	19.0
L. amazonensis 4G1	30.4
L. amazonensis 4G2	20.1
L. amazonensis 4G3	26.4
L. amazonensis 4G4	24.6

Figure S2. (A). LeishIF4G5 sequence showing the putative YxxxxL motives. The YxxxxL motif is required to bind with LeishIF4E protein, whereby Y refers to Tyrosine, X refers to any amino acid and L refers to Leucine. (B) The **presence of a MIF4G domain in LeishIF4G5.** LeishIF4G5 sequence was scanned using InterPro online tool InterPro (ebi.ac.uk) **(C) Sequence similarity of *L. amazonensis* LeishIF4G5 with various other *Leishmania* and mouse paralogs.** The table shows percent similarities between the LeishIF4G5 from *L. mexicana* and different *Leishmania* LeishIF4Gs, along with the *Mus Musculus* eIF4G. Percent similarities were generated by EMBOSS needle (https://www.ebi.ac.uk/Tools/psa/emboss_needle/).

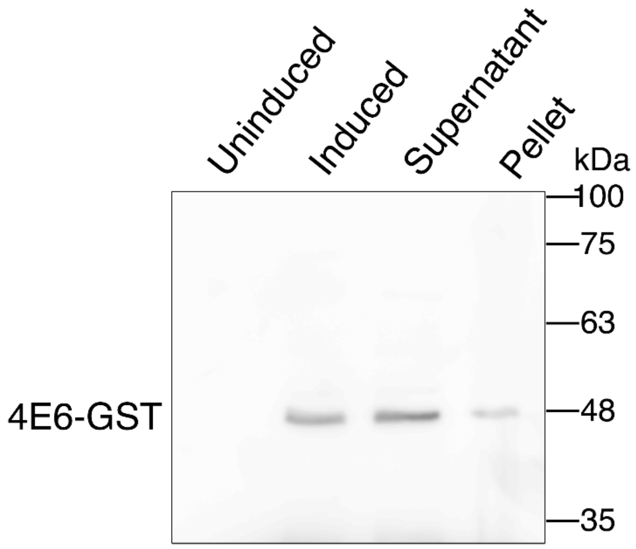

Figure S3. Expression of recombinant GST tagged LeishIF4E-6 in *E.coli*. Full-length Leish4E-6 tagged with GST was expressed in *E. coli* BL-21 cells. Aliquots of total extracts derived from Uninduced, induced with 0.5 mM IPTG (Induced), and aliquots from the soluble supernatant and Pellet fractions were separated over 10% SDS-PAGE gel and subjected to western analysis with specific antibodies against GST.

A

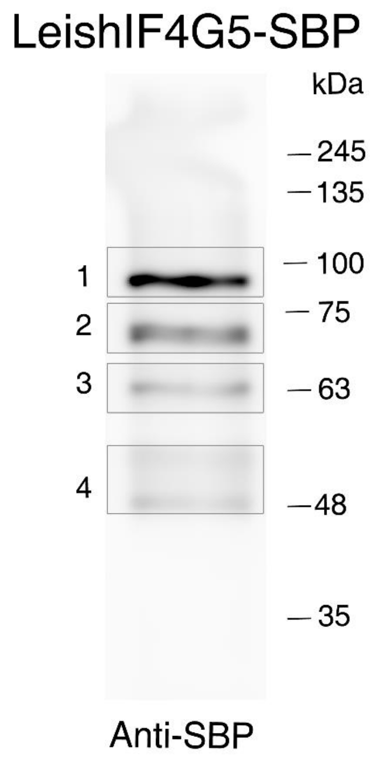

B

Band number	Number of peptides associated with LeishIF4G5
1	48
2	40
3	18
4	23

Figure S4. **LeishIF4G-5 is susceptible to proteolytic cleavage**. (**A)** Lysates of *L. amazonensis* expressing SBP tagged LeishIF4G-6 were purified over Streptavidin Sepharose beads and the eluted fractions were separated over 10% SDS-PAGE that were processed for western analysis using monoclonal antibodies raised against SBP tag. The bands that interacted with the antibodies were cut from a parallel gel and further subjected to Mass Spectrometry analysis. **(B)** Table shows the number of peptides obtained in the Mass Spectrometry analysis of each band. The original Mass Spectrometry data are given in Table S3.
