# Supplementary material for: Characterization of an Atypical eIF4E Ortholog in Leishmania, LeishIF4E-6
Source: Int J Mol Sci. 2021 Nov 24;22(23):12720. doi: 10.3390/ijms222312720 (PMC8657474; doi:10.3390/ijms222312720)
Supplement: Supplementary file 1 [file ijms-22-12720-s001.zip › ijms-1437572-supplementary/SUP_Captions.pdf]

Figure S1. (A) Alignment of *L. amazonensis* eIF4E-6 with *T. brucei* counterpart. The eIF4E-6 sequences from *L. amazonensis* and *T. brucei were* subjected to sequence alignment using Jalview 2.11.1.4. Dashes represent spaces that were inserted to allow better alignment. Conserved Tryptophan, Tyrosine and Phenylalanine residues involved in the cap-binding activity are highlighte in red boxes. (B) **Sequence similarity of *L. amazonensis* LeishIF4E-6, with various other paralogs and mammalian eIF4E-1.** The table shows percent similarities between the LeishIF4E-6 and different *Leishmania* LeishIF4Es, the *Mus Musculus* eIF4E and *T. brucei* TbIF4E-6. Percent similarities were generated by EMBOSS needle (https://www.ebi.ac.uk/Tools/psa/emboss_needle/).

Figure S2. (A). LeishIF4G5 sequence showing the putative YxxxxL motives. The YxxxxL motif is required to bind with LeishIF4E protein, whereby Y refers to Tyrosine, X refers to any amino acid and L refers to Leucine. (B) The **presence of a MIF4G domain in LeishIF4G5.** LeishIF4G5 sequence was scanned using InterPro online tool InterPro (ebi.ac.uk) **(C) Sequence similarity of *L. amazonensis* LeishIF4G5 with various other *Leishmania* and mouse paralogs.** The table shows percent similarities between the LeishIF4G5 from *L. mexicana* and different *Leishmania* LeishIF4Gs, along with the *Mus Musculus* eIF4G. Percent similarities were generated by EMBOSS needle (https://www.ebi.ac.uk/Tools/psa/emboss_needle/).

Figure S3. Expression of recombinant GST tagged LeishIF4E-6 in *E.coli*. Full-length Leish4E-6 tagged with GST was expressed in *E. coli* BL-21 cells. Aliquots of total extracts derived from Uninduced, induced with 0.5 mM IPTG (Induced), and aliquots from the soluble supernatant and Pellet fractions were separated over 10% SDS-PAGE gel and subjected to western analysis with specific antibodies against GST.

Figure S4. **LeishIF4G-5 is susceptible to proteolytic cleavage**. (**A)** Lysates of *L. amazonensis* expressing SBP tagged LeishIF4G-6 were purified over Streptavidin Sepharose beads and the eluted fractions were separated over 10% SDS-PAGE that were processed for western analysis using monoclonal antibodies raised against SBP tag. The bands that interacted with the antibodies were cut from a parallel gel and further subjected to Mass Spectrometry analysis. (**B)** Table shows the number of peptides obtained in the Mass Spectrometry analysis of each band. The original Mass Spectrometry data are given in Table S3.
